# *Calculus Bovis* ameliorates primary sclerosing cholangitis via a dual-pronged mechanism restoring bile acid and lipid homeostasis in the gut-liver axis

**DOI:** 10.1186/s13020-026-01441-w

**Published:** 2026-06-08

**Authors:** Xuepeng Gong, Yufei Chen, Tinghui Zhao, Ninghong Li, Guangjie Yang, Lihui Qiu, Zaoqin Yu, Dong Liu, Dong Xiang

**Affiliations:** 1 Department of Pharmacy, Tongji Hospital, Tongji Medical College, Huazhong University of Science and Technology, Wuhan, 430030 Hubei China; 2 Department of Pharmacy, Wuhan Mental Health Center, Wuhan, 430030 Hubei China; 3 Department of Pharmacy, The Third Affiliated Hospital of Nanchang University, Nanchang, 330008 Jiangxi China

**Keywords:** Primary sclerosing cholangitis, *Calculus Bovis*, Bile acid profile, Lipid metabolism, Gut-liver axis

## Abstract

**Objective:**

Primary sclerosing cholangitis (PSC) is a progressive cholestatic liver disease lacking FDA-approved therapy. *Calculus Bovis* (CB), a traditional medicine derived from animal gallstones, has been historically used for treating hepatobiliary diseases, but its therapeutic potential and mechanisms in PSC remain unexplored. This study aimed to investigate the efficacy of CB in an experimental PSC model and elucidate its underlying mechanisms.

**Methods:**

A PSC mouse model was induced by a 0.1% 3,5-diethoxycarbonyl-1,4-dihydrocollidine (DDC) diet for 4 weeks. Mice were treated with CB (50,100, 150 mg/kg/day) or ursodeoxycholic acid (UDCA, 100 mg/kg/day). Liver injury, fibrosis, intestinal barrier integrity, bile acid (BA) profiles, and lipid levels were assessed. Hepatic and intestinal gene/protein expression related to BA and lipid metabolism was analyzed. Integrated transcriptomics, network pharmacology, and *in vitro* serum pharmacology were employed to elucidate the underlying mechanisms.

**Results:**

CB administration significantly alleviated liver injury, fibrosis, and intestinal barrier damage in DDC-induced mice. It restored BA homeostasis across the gut-liver axis, normalizing aberrant BA profiles in serum and liver while increasing BA excretion in feces. CB also ameliorated dyslipidemia, reducing hepatic and serum lipid levels. Mechanistically, CB and its bioactive BA components exerted their effects through a dual-pronged mechanism: (1) activation of the SIRT1-PGC-1α axis to transcriptionally upregulate the expression of nuclear receptors FXR and PPARα in the liver and intestine, and (2) direct ligand-dependent activation of FXR and PPARα protein functions. This concerted activation enhanced the transcription of genes involved in BA detoxification, transport, and fatty acid β-oxidation. Inhibition of SIRT1 or antagonism of FXR/PPARα attenuated these protective effects *in vitro.*

**Conclusion:**

CB attenuates experimental PSC by modulating BA and lipid homeostasis via the gut-liver axis, mediated through a novel dual mechanism involving SIRT1-PGC-1α pathway activation and direct receptor agonism. These findings not only highlight CB as a promising multi-target agent for PSC treatment, but also provide novel insights into the therapeutic modulation of metabolism in the gut-liver axis.

**Supplementary Information:**

The online version contains supplementary material available at 10.1186/s13020-026-01441-w.

## Introduction

Primary sclerosing cholangitis (PSC) is a chronic, progressive cholestatic liver disease characterized by inflammation and fibrosis of the intra- and extrahepatic bile ducts, ultimately progressing to cirrhosis, liver failure, and an elevated risk of cholangiocarcinoma [1]. Despite its strong association with inflammatory bowel disease, particularly ulcerative colitis, the precise etiology and pathogenesis of PSC remain poorly understood [2]. Epidemiological trends indicate a rising global prevalence, especially in Northern Europe and North America (0.78–31.7 per 100,000), underscoring its growing clinical burden [3, 4]. Currently, there are no specific therapeutic drugs approved by the U.S. Food and Drug Administration (FDA) for PSC [4]. Standard therapies such as ursodeoxycholic acid (UDCA) may improve biochemical parameters in some patients but have not been proven to delay disease progression or improve survival [4]. For end-stage patients, liver transplantation remains the only curative option, yet post-transplant recurrence poses a significant challenge [4]. Therefore, the exploration of novel and effective treatment strategies is an urgent clinical priority.

Emerging evidence highlights the “gut-liver axis” as central to the pathophysiology of PSC [5]. Bile acids (BAs), crucial for lipid digestion and absorption, also function as signaling molecules regulating metabolic, inflammatory, and immune homeostasis in the liver and intestine [6, 7]. In PSC, dysregulation of BA synthesis, secretion, intestinal reabsorption, and enterohepatic circulation leads to hepatic accumulation of hydrophobic, cytotoxic BAs. This directly injures cholangiocytes, induces oxidative stress and inflammation, and drives fibrogenesis [8–10]. Concurrently, BA metabolic dysregulation forms a vicious cycle with impaired intestinal barrier function and microbial dysbiosis, often accompanied by significant lipid metabolism abnormalities [11, 12]. Consequently, restoring BA and lipid metabolic homeostasis within the gut-liver axis has emerged as a promising therapeutic strategy for PSC. Nuclear receptors such as the Farnesoid X Receptor (FXR) and Peroxisome Proliferator-Activated Receptor α (PPARα) serve as master transcriptional regulators of BA synthesis, transport, and fatty acid β-oxidation, acting as critical hubs in maintaining this homeostasis [13, 14].

Targeting individual receptors, such as the FXR agonist obeticholic acid or PPARα agonists like fibrates, has yielded limited clinical progress in PSC, underscoring the challenges of single-target therapies for this complex, multifactorial disease [15, 16]. In this context, traditional medicines featuring multi-component, multi-target synergistic effects present a novel approach for systemically correcting metabolic network imbalances. Calculus Bovis (CB), a traditional animal-derived medicine with a long history of use in treating hepatobiliary disorders, first documented in *Shennong’s Classic of Materia Medica*, is the gallstone of water buffalo or cattle [17]. Originating in China, the medicinal use of CB has spread with traditional medicine and gained wide application in East and Southeast Asian regions such as China, Korea, Japan, and Singapore. In traditional Chinese medicine theory, CB has been consistently used to treat conditions related to the hepatobiliary system, such as jaundice, high fever, and convulsions. However, the modern pharmacological mechanisms by which CB might treat PSC remain completely unexplored. It is particularly crucial to investigate whether CB can exert therapeutic effects by modulating the gut-liver axis as an integrated network and whether its action involves more upstream energy and metabolic sensing pathways.

This study was designed to systematically investigate the therapeutic efficacy of CB in an experimental PSC model and elucidate its underlying mechanisms. Using a combination of *in vivo* and *in vitro* approaches, we found that CB and its bioactive BA components synergistically enhance FXR and PPARα signaling through a dual mechanism involving SIRT1-PGC-1α-mediated transcriptional upregulation and direct receptor activation. This concerted action promotes the detoxification and excretion of toxic BAs, improves fatty acid oxidation, and restores intestinal barrier integrity. This study not only aims to provide preclinical evidence for CB as a potential therapeutic strategy for PSC, but also seeks to reveal a novel natural‑medicine‑mediated pathway for restoring gut-liver axis homeostasis.

## Materials and methods

### Chemicals and reagents

The feed containing 0.1% DDC (purity > 98%, Aladdin, Shanghai) was customized by Jiangsu Xietong Pharmaceutical and Biological Engineering Co., Ltd. (Nanjing, China). Aspartate aminotransferase (AST, C010-2-1), alanine aminotransferase (ALT, C009-2-1), alkaline phosphatase (ALP, A059-2-2), total bilirubin (TBIL, C019-1-1), direct bilirubin (DBIL, C019-2-1), glutathione (GSH, A006-2-1), total cholesterol (TCHO, A111-1-1), triglyceride (TG, A110-1-1), high-density lipoprotein cholesterol (HDLC, A112-1-1) and low-density lipoprotein cholesterol (LDLC, A113-1-1) kits were obtained from the Nanjing Jiancheng Institute of Biotechnology (Nanjing, China). Malondialdehyde (MDA, S0131S) and free fatty acid (FFA, S0215S) kits were obtained from Beyotime Biotechnology Co., LTD (Shanghai, China). Hydroxyproline (HYP, BC0255) was obtained from Beijing Solarbio Science & Technology Co., LTD (Beijing, China). SIRT1 inhibitor SIRT1-IN-1 (#HY136199) and FXR antagonist Z-guggulsterone (#HY-110066) were purchased from Med Chem Express (Shanghai, China). PPARα antagonist MK-886 (#S80147) and bilirubin (#V99121) were purchased from Orileaf (Shanghai, China). Manufacturer details of BAs, primers, and antibodies are provided in the supplementary materials.

### Quality control of CB

The natural CB used in this study was purchased from Wuhan Zeyou Biotechnology Co., Ltd. (Batch No. 20230703). Quality assessment was conducted in accordance with the Chinese Pharmacopoeia (2025 edition) guidelines. Visual inspection and authenticity tests were performed, with all results meeting the stipulated standards. The bilirubin content was determined using high-performance liquid chromatography (HPLC) and found to be 272 mg/g (representative chromatogram in Fig. S1A). Given that BAs represent the principal active constituents of CB, liquid chromatography-tandem mass spectrometry (LC–MS/MS) was employed to identify the types and quantify the content of BAs. Representative chromatograms are shown in Fig. S1A, with detailed quantitative results summarized in Supplementary Table S1. The total content of conjugated BAs, including taurocholate (TCA), taurodesoxycholate (TDCA), glycocholic acid (GCA), and glycodesoxycholic acid (GDCA), was 51.3 mg/g. The total content of free BAs, including cholic acid (CA) and chenodeoxycholic acid (CDCA), was 22.3 mg/g. Consequently, the contents of conjugated BAs, unconjugated BAs, and bilirubin all met the requirements of the Chinese Pharmacopoeia (conjugated BAs > 4%, unconjugated BAs < 8%, and bilirubin > 25%), confirming the suitability of the CB material for subsequent experimental studies. To further characterize the chemical profile of CB, a fingerprint was acquired using ultra-high-performance liquid chromatography coupled with quadrupole-Orbitrap mass spectrometry (UHPLC-Q-Orbitrap MS) (Fig. S1B). The fingerprint illustrates the complex composition of CB, with major peaks preliminarily identified as various BAs and amino acids (Supplementary Table S2). This fingerprint can serve as a reference for quality control and the exploration of bioactive components.

### Animal experiments

All animal experimental procedures were approved by the Experimental Animal Welfare and Ethics Committee of Tongji Hospital, Tongji Medical College, Huazhong University of Science and Technology (Approval number: TJH-202311030) and were conducted in accordance with the National Institutes of Health Guide for the Care and Use of Laboratory Animals. Male C57BL/6 mice (6–8 weeks old) were purchased from Hunan SJA Laboratory Animal Co., Ltd. (Changsha, China). All animals were housed under specific-pathogen-free (SPF) conditions in a controlled environment (20–24  C, 60–70% relative humidity) with a 12-h light–dark cycle and free access to food and water. Animals were humanely euthanized at the experimental endpoint. Anesthesia was induced and maintained with isoflurane before blood collection via orbital plexus. Following blood collection, mice were sacrificed by cervical dislocation under deep anesthesia to minimize suffering. All personnel involved were certified and adhered to the 3R principles (Replacement, Reduction, and Refinement).

After one week of acclimatization, mice were randomly divided into six groups (n = 6 per group): (1) Control + Veh group: fed a normal diet and administered vehicle (0.5% sodium carboxymethyl cellulose, CMC-Na) by oral gavage once daily; (2) DDC + Veh model group: fed a 0.1% DDC diet and administered vehicle; (3–5) DDC + CB groups: fed a 0.1% DDC diet and administered CB at 50,100, or 150 mg/kg/day, respectively; (6) DDC + UDCA group: fed a 0.1% DDC diet and administered UDCA at 100 mg/kg/day. Based on our preliminary experiments and published literature [18, 19] on its hepatoprotective effects, the CB doses in mice (50, 100, 150 mg/kg/day) were selected. According to the FDA-recommended body surface area conversion standard, these doses correspond to human equivalent doses of approximately 4.06–12.17 mg/kg/day (calculated based on a 60 kg adult). The administration volume was 10 mL/kg body weight. Treatments lasted for 4 weeks. At the end of the experiment, mice were anesthetized after a 12-h fast. Blood was collected from the orbital plexus, and serum was separated by centrifugation (3500 rpm for 10 min at 4 °C) for biochemical and BA profile analyses. Liver, ileum, and fecal samples were rapidly collected, snap-frozen in liquid nitrogen, and stored at − 80 °C for subsequent analysis. A portion of the liver tissues was fixed in 10% neutral buffered formalin for histopathological examination. For transcriptomic and BA metabolomic analyses, samples from the Control + Veh, DDC + Veh, and DDC + CB (150 mg/kg) groups were utilized.

### Preparation of drug-containing serum and artificial bile acid mixture (ABA)

Sprague–Dawley (SD) rats (6–8 weeks old, male) were obtained from the same vendor and housed under identical SPF conditions. After one week of adaptive feeding, rats were randomly divided into two groups (n = 15 per group): the blank control group and the CB administration group. The CB group received CB (375 mg/kg) by oral gavage twice daily for three consecutive days, while the control group received an equal volume of vehicle. Rats were chosen over mice for serum preparation due to their larger blood volume, which ensured sufficient and consistent serum supply for the multiple *in vitro* experiments, thereby minimizing batch-to-batch variability. The dose (375 mg/kg) was calculated by body surface area conversion from the effective mouse dose (150 mg/kg), which is approximately 5 times the mouse dose. Thirty minutes after the last administration (a time point corresponding to the approximate Tmax of major BA components based on preliminary experiments), blood was collected from the abdominal aorta under anesthesia. The blood was allowed to clot at room temperature for 2 h and then centrifuged at 3500 rpm for 10 min. The CB-containing serum (CB-S) or control serum (Con-S) were collected, inactivated (56 °C for 30 min), passed through a 0.22 μm sterile filter, and stored at − 80 °C for subsequent cell experiments.

The BA concentration in CB-S and Con-S was determined using the LC–MS/MS method described in Sect. “BA profile analysis”. The resulting composition of blood-entry BAs is provided in Supplementary Table 1. The ten most abundant BAs (HDCA, DCA, CA, CDCA, 12-KCDCA, TCA, GCA, 7-KDCA, 12-KDCA, and LCA), accounting for > 80% of total BA content, were reconstituted in control rat serum and serum-free medium at the same concentrations as detected in 20% CB-S. Bilirubin was dissolved in DMSO and diluted to the same concentration as in 20% CB-S (17.52 μmol/L) either in control serum or in serum-free medium.

### Cell culture and treatment

Human hepatocellular carcinoma cells (HepG2) and human colorectal adenocarcinoma cells (Caco-2) were obtained from the American Type Culture Collection (ATCC). Cells were cultured in Dulbecco’s Modified Eagle Medium/Nutrient Mixture F-12 (DMEM/F-12) supplemented with 10% fetal bovine serum (FBS), 1% penicillin/streptomycin (P/S), and 1% non-essential amino acids (NEAA) solution. Cells were maintained at 37 °C in a humidified atmosphere with 5% CO₂, and the culture medium was changed every 2–3 days. Cells were seeded in 6-well plates at a density of 2 × 10^5^ cells per well. After reaching approximately 80% confluence, the culture medium was replaced with fresh medium containing 1% FBS for synchronization for 12 h. Cells were then treated as follows: To establish an *in vitro* cellular injury model, cells were incubated with 100 μM DDC for 24 h (a concentration based on cytotoxicity assays, see Fig. S5A). Concurrently, cells were co-treated with different concentrations (5, 10, 20%) of CB-S or Con-S. For pharmacological intervention studies, cells were pre-treated for 2 h with the SIRT1 inhibitor SIRT1-IN-1 (2.0 μM), the FXR antagonist Z-guggulsterone (10 μM), or the PPARα antagonist MK-886 (10 μM) before the addition of DDC and CB-S. The concentrations were selected based on cell viability (CCK-8) assays (Fig. S5A) and previously reported effective inhibitory concentrations [20–23].

For validation experiments of the ABA mixture and bilirubin, cells were seeded in six-well plates and synchronized as described above, followed by exposure to 100 μM DDC. Concurrently, cells were treated with: (1) 20% Con-S; (2) 20% CB-S; (3) 20% Con-S supplemented with ABA or bilirubin at the same concentrations as in 20% CB-S; (4) ABA or bilirubin alone (in serum-free medium). The solvent control (DMSO, < 0.1%) was included where applicable. To validate the direct receptor activation by key BA constituents of CB, separate groups of cells were treated with individual BAs, including CA, CDCA, deoxycholic acid (DCA), and hyodeoxycholic acid (HDCA), at a concentration of 50 μM. After 24 h of incubation, cells were harvested for RNA and protein extraction.

### Biochemistry and histological analysis

Serum levels of ALT, AST, ALP, TBIL, DBIL, FFA, TCHO, TG, HDLC, and LDLC, along with hepatic levels of MDA, GSH, FFA, TCHO, and TG, as well as intestinal levels of TCHO and TG, were measured using commercial kits according to the manufacturer's instructions on a multifunctional microplate reader (Synergy2, BioTek, USA). For histological analysis, liver tissues were fixed in 10% formalin, embedded in paraffin, and sectioned at 4 μm thickness. Intestinal tissues were similarly fixed, embedded, and sectioned, followed by H&E staining to evaluate villus morphology and inflammatory infiltration. Sections were stained with Hematoxylin and Eosin (H&E) for general morphology assessment. Hepatic lipid accumulation was assessed using frozen liver sections stained with Oil Red O. All sections were observed and imaged under an optical microscope (Nikon, Tokyo, Japan). The positive area of Oil Red O staining was quantified using ImageJ software (National Institutes of Health, USA).

### BA profile analysis

The LC–MS/MS analytical method targeting BAs has been slightly revised based on our previously published article [24]. A 50 μL aliquot of serum or homogenate supernatant from liver, ileum, or fecal samples was added to 150 μL of methanol solution containing the internal standard chenodeoxycholic acid-d4 (CDCA-d4), followed by the addition of 50 μL of pure methanol. The mixture was vortexed for 5 min and centrifuged at 12,000 × *g* for 10 min. Subsequently, 200 μL of the supernatant was collected and dried under vacuum. The dried residue was reconstituted with methanol, acetonitrile, and water to a final volume of 180 μL, and the resulting supernatant was subjected to injection analysis. BA analysis was performed using an LC-20AD HPLC system coupled in tandem to an AB Qtrap 5500 mass spectrometer (AB Sciex, USA). Chromatographic separation of 50 types of BAs was achieved on a Welch Ultimate XS-C18 column (5 μm, 4.6 mm × 150 mm) with a mobile phase consisting of (A) 10 mM ammonium formate containing 0.1% formic acid and (B) methanol–acetonitrile (50:50, v/v) containing 0.1% formic acid. The gradient elution program was as follows: 0–1 min, 0–30% B; 1–26 min, 30–72% B; 26–38 min, 72–98% B; 38–42 min, 98% B; 42–42.10 min, 98% B; 42.10–45 min, 98–30% B. Mass spectrometric detection was conducted using electrospray ionization (ESI) in negative ion mode. All BA information and full names of BA abbreviations are listed in Supplementary Table S3.

### Immunofluorescence staining

After deparaffinization, rehydration, and antigen retrieval of paraffin-embedded liver tissue sections, the sections were incubated with 3% hydrogen peroxide solution to block endogenous peroxidase activity, and then blocked with 10% goat serum at room temperature for 30 min. The sections were incubated with primary antibody against CK-19 (1:250) or Cleaved Caspase-3 (1:100) at 4 °C overnight. For intestinal ZO-1 immunofluorescence, paraffin-embedded ileal sections were subjected to the same deparaffinization, antigen retrieval, and blocking steps. The sections were then incubated with a primary antibody against ZO-1 (1:200) overnight at 4 °C. After washing, sections were incubated with a fluorescently labeled secondary antibody at room temperature for 1 h in the dark. After nuclear counterstaining with DAPI, fluorescent microscopy examination was performed using a microscope (Olympus BX51, Japan). The fluorescence-positive area was quantified using ImageJ software.

### RNA sequencing and qPCR

Total RNA was extracted from liver tissues using TRIzol^®^ (Life Technologies, USA) and quantified by NanoDrop 2000 spectrophotometer (Thermo Scientific, USA). Library construction was performed on samples whose RNA purity and integrity were detected by Agilent 2100 Bioanalyzer (Agilent Technologies, USA). After mRNA isolation, cDNA synthesis, terminal repair, A-tailing, adapter ligation, purification, and PCR amplification, sequencing was performed on the Illumina NovaSeq 6000 platform. Sequencing reads were analyzed using DESeq2, with |Fold-Change|> 2 and adjusted p-value < 0.05 set as the threshold for significant differential expression. Functional enrichment analysis was performed using clusterProfiler for annotation of Gene Ontology (GO) and Kyoto Encyclopedia of Genes and Genomes (KEGG) pathways (FDR < 0.05). Gene set enrichment analysis (GSEA) was conducted via the OmicShare website platform (https://www.omicshare.com/tools/home/report/report _gsea.html).

Following total RNA extraction and quantification, cDNA was synthesized using PrimeScript™ RT Master Mix (Takara, Tokyo, Japan). qRT-PCR reactions were carried out with TB Green^®^ Premix Ex Taq^™^ II (Takara, Tokyo, Japan) by StepOne Plus™ Real-Time PCR System (Applied Biosystems, USA). The relative mRNA expression levels were calculated using the 2^ −△△Ct^ method and normalized to the housekeeping gene *Hprt1* or *GAPDH*. The sequences of all primers used are listed in Supplementary Table S4.

### Western blot

Liver tissues or cultured cells were lysed in RIPA buffer (P0038, Beyotime, China) supplemented with protease and phosphatase inhibitors. Protein concentration was quantified using a BCA assay kit (P0012, Beyotime, China). Equal amounts of protein (20–40 μg) were separated by 8–15% SDS-PAGE and electrophoretically transferred onto polyvinylidene difluoride (PVDF) membranes (Millipore, MA, USA). The membranes were blocked with 5% skimmed milk in 0.1% TBST and then incubated overnight with specific primary antibodies at 4 °C on a shaker. After washing, the PVDF membranes were incubated with HRP-conjugated secondary antibodies at room temperature for 1 h. Protein bands were visualized using a chemiluminescence imaging system (G: BOX Chemi XRQ, Syngene, UK), and band intensities were quantified using ImageJ software, normalized to GAPDH. All antibody information is listed in Supplementary Table S5.

### Network pharmacology and molecular docking

To elucidate the potential mechanisms of CB, a network pharmacology approach was conducted based on its blood-entering components. The BA constituents detected in the serum after CB administration (as described in Sect. “Preparation of drug-containing serum and artificial bile acid mixture (ABA)”) were defined as the active compounds of CB for target prediction. Potential targets of the active compounds were predicted through SwissTargetPrediction (http://www.swisstargetprediction.ch) with a credibility score threshold set at Probability > 0.1. Disease-related targets for PSC were retrieved from GeneCards (https://www.genecards.org), OMIM (https://www.omim.org), DisGeNET (https://disgenet.com), and CTD (https://ctdbase.org). The relevance score thresholds for screening disease-related targets in the GeneCards, DisGeNET, and CTD databases were set to > 10, > 0.01, and > 60, respectively. The compounds and their targets were imported into Cytoscape 3.8.2 to construct a compound-target network. The protein–protein interaction (PPI) network was constructed and visualized using the STRING database (https://string-db.org) with a minimum required interaction score set to 0.7 (medium confidence) and Cytoscape 3.8.2. GO and KEGG enrichment analyses for the overlapping targets were performed using the DAVID database (https://david.ncifcrf.gov/).

For molecular docking, the 3D structures of target proteins FXR (3BEJ) and PPARα (6KAX) were downloaded from the RCSB PDB (http://www.rcsb.org/). The structures of key BA components were obtained from the PubChem database (https://pubchem.ncbi.nlm.nih.gov/). Docking simulations were performed using AutoDock 4.2.6, and the results were visualized with PyMOL 2.3.0.

### Statistical analysis

All data were analyzed and visualized using GraphPad Prism 10.0 and were presented as the mean ± standard error of the mean (SEM). Unpaired Student’s t-test was used to assess significant differences between the means of two groups, while one-way analysis of variance (ANOVA) followed by Tukey's post hoc test was employed to determine statistical significance for experiments involving more than two groups. A p-value < 0.05 was considered statistically significant.

## Results

### CB ameliorates DDC-induced liver injury, fibrosis, and intestinal barrier damage

**Fig. 1:**
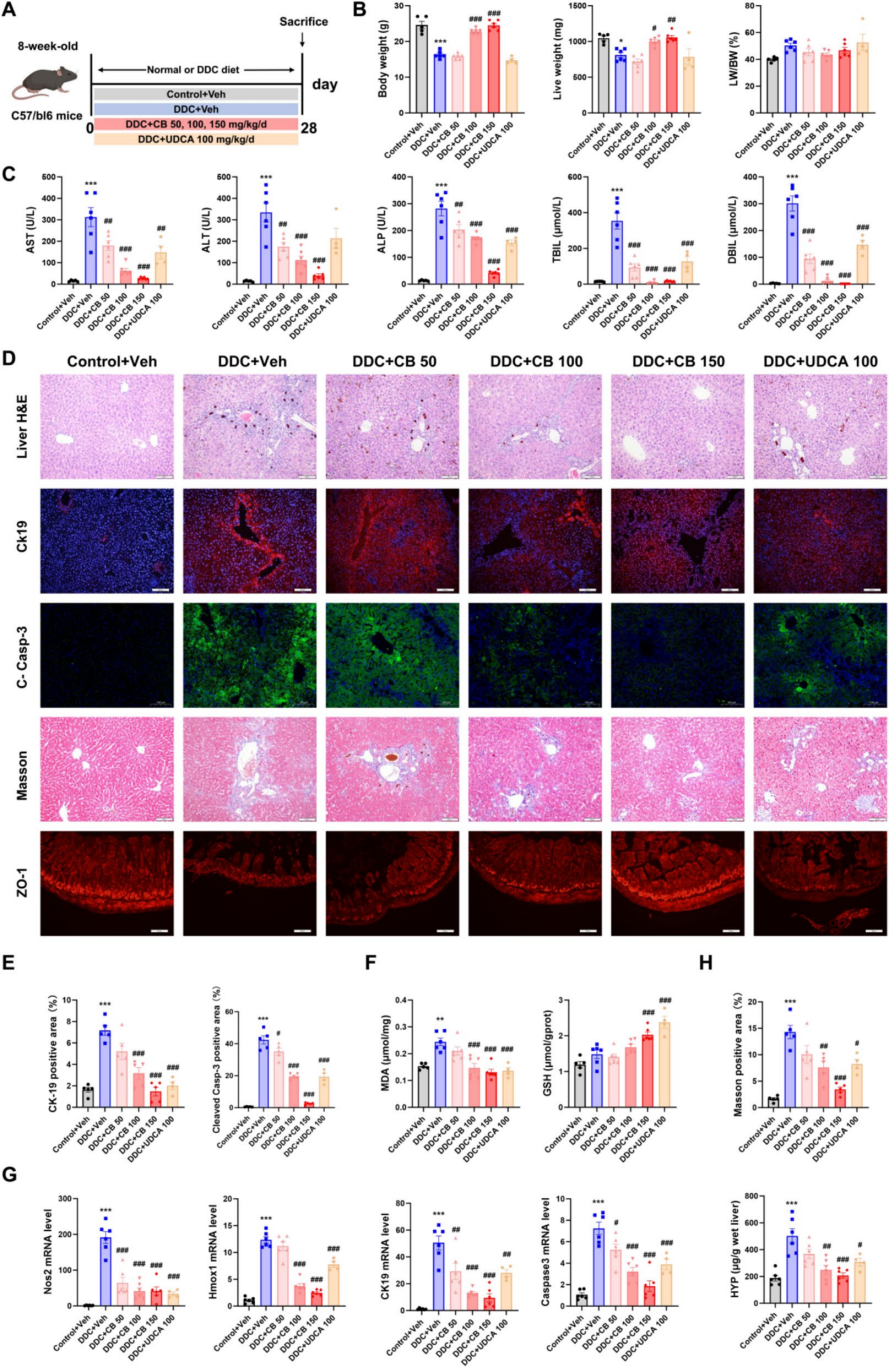
CB ameliorates liver injury, fibrosis, and intestinal barrier damage in a murine model of PSC. **A** Schematic overview of the experimental design. Mice were fed a normal or 0.1% DDC diet for 28 days with daily intragastric administration of vehicle (Veh), CB (50, 100, or 150 mg/kg), or UDCA (100 mg/kg). **B** Body weight, liver weight, and liver-to-body weight ratio (LW/BW). **C** Serum levels of ALT, AST, ALP, total bilirubin (TBIL), and direct bilirubin (DBIL). **D** Representative images of liver sections stained with H&E, immunofluorescence for CK19 and cleaved caspase‑3 (C‑Casp3), Masson’s trichrome, and ileal sections immunostained for ZO‑1. Scale bars: 50 or 100 μm. **E** Quantitative analysis of the positive staining area for CK19 and C-Casp3. **F** Hepatic levels of malondialdehyde (MDA) and glutathione (GSH). **G** Hepatic mRNA expression of *Nos2*, *Hmox1*, *CK19*, and *Caspase-3*, normalized to *Hprt1*. **H** Quantitative analysis of the fibrosis area (Masson’s staining) and hepatic hydroxyproline content. Data are presented as mean ± SEM (n = 4–6). ^*^p < 0.05, ^**^p < 0.01, ^***^p < 0.001 *vs*. Control + Veh; ^#^*p* < 0.05, ^##^*p* < 0.01, ^###^*p* < 0.001 *vs*. DDC + Veh

To evaluate the therapeutic potential of CB in PSC, we utilized a mouse model of PSC induced by a 0.1% DDC-supplemented diet for 28 days. Throughout the dietary intervention, mice were treated with either the vehicle or different doses of CB (Fig. 1A). Compared to the DDC model mice, CB treatment normalized the reduction in body and liver weights (Fig. 1B) and dose-dependently decreased serum levels of AST, ALT, ALP, TBIL, and DBIL, the key markers of liver function (Fig. 1C). CB remarkably ameliorated DDC-induced macroscopic and pathological changes of liver, as evidenced by restored liver color, and reduced necrosis as well as inflammatory cell infiltration (Fig. 1D and Fig. S2A). Moreover, CB significantly reduced bile duct proliferation and apoptosis as indicated by CK19 (a marker for bile duct proliferation) and Cleaved Caspase-3 (a marker for apoptosis) immunofluorescence staining (Fig. 1D, E). Additionally, CB decreased the level of the pro-oxidant MDA and increased the level of anti-oxidant GSH in DDC mice (Fig. 1F). The increased mRNA expression of Hmox1 and Nos2 (two oxidative stress markers), as well as CK19 and Caspase-3, was suppressed by CB treatment, further supporting the alleviation of oxidative stress, bile duct proliferation, and apoptosis by CB (Fig. 1G). Moreover, Masson staining and hepatic hydroxyproline quantification confirmed that CB significantly reduced hepatic collagen deposition and fibrosis progression (Fig. 1D, H).

Given that intestinal barrier dysfunction and subsequent bacterial translocation contribute to PSC pathogenesis, we further examined the effects of CB on the gut. H&E and immunofluorescence staining of intestinal tissue showed that CB effectively improved villous atrophy or loss, inflammatory infiltration, and loss of the tight junction protein ZO-1 in DDC mice (Fig. 1D and Fig. S2A, S2B). Correspondingly, CB upregulated mRNA expression of tight junction-related genes, *Tjp1*, *Oldn3*, and *Ocln*, indicating a restorative effect on intestinal barrier integrity (Fig. S2C). Notably, high-dose CB showed a more pronounced ameliorative effect than the UDCA (positive control) group in several parameters (Fig. 1 and Fig. S2A-C). Collectively, these results indicate that CB alleviates DDC-induced liver injury, hepatic fibrosis, and intestinal barrier damage.

Critically, we observed no adverse effects in mice treated with CB. Normal mice treated with a high dose of CB for 28 days showed no abnormalities in the heart, liver, spleen, lung, kidney, ileum, or colon (Fig. S2D). Body, liver, and spleen weights, as well as the serum levels of AST, ALT, urea nitrogen, and creatinine (liver and kidney function), all remained unchanged (Fig. S2E, S2F), suggesting that CB is safe.

### Transcriptome analysis and network pharmacology link the therapeutic effects of CB to the modulation of BA and lipid metabolism

**Fig. 2:**
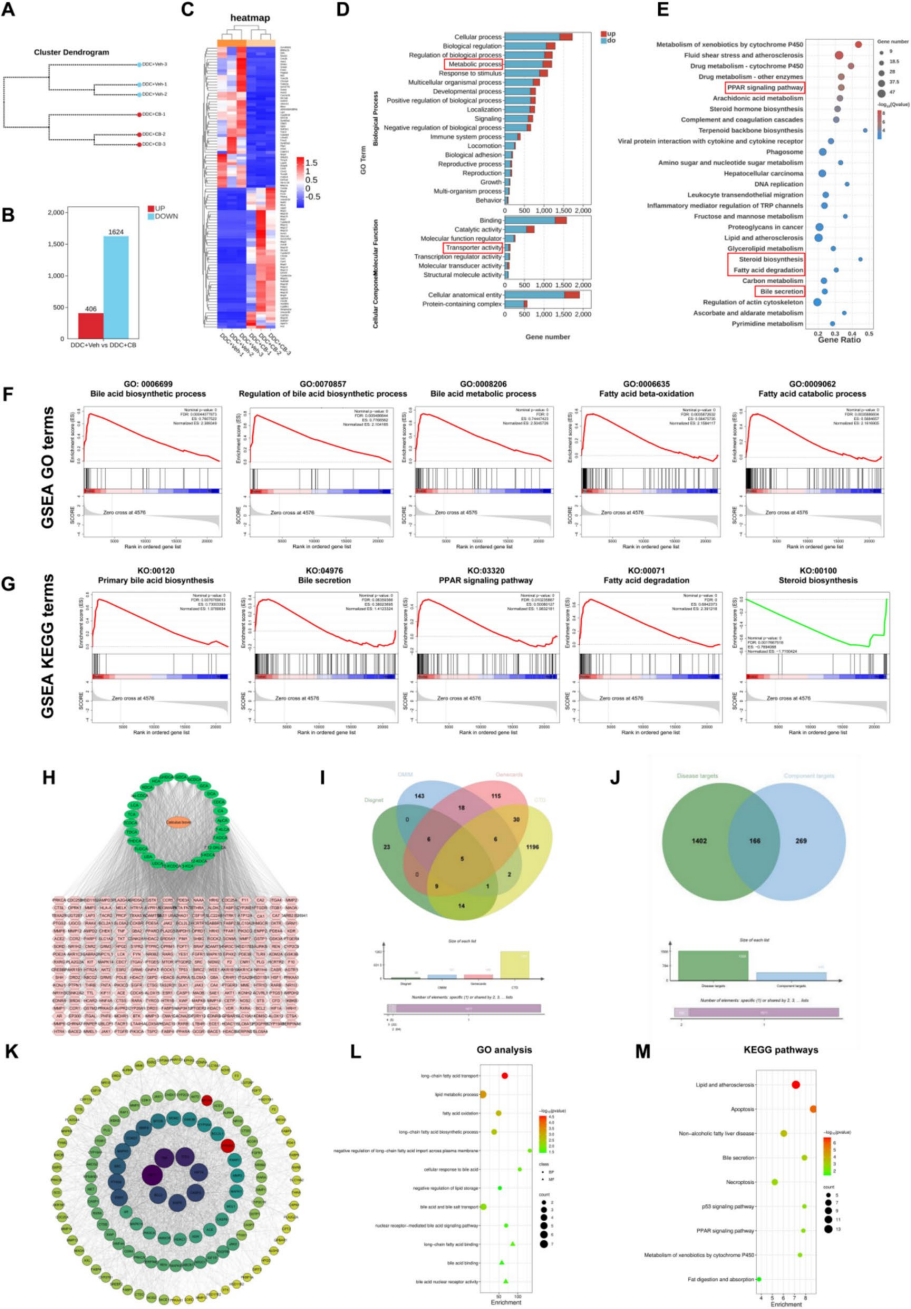
Integrated transcriptomics and network pharmacology link the therapeutic effects of CB to the modulation of BA and lipid metabolism. **A** Intergroup clustering diagram between DDC + Veh and DDC + CB groups. **B** The number of significantly upregulated and downregulated genes in the DDC + CB group compared to the DDC + Veh group. **C** Heatmap of the top 100 differentially expressed genes (DEGs). **D**, **E** Gene Ontology (GO) biological process terms **D** and Kyoto Encyclopedia of Genes and Genomes (KEGG) pathways **E** were significantly enriched among the DEGs. **F**, **G** Gene Set Enrichment Analysis (GSEA) plots showing significant enrichment of GO terms **F** and KEGG pathways **G** related to BA and lipid metabolism after CB treatment. **H** Compound-target network of blood-entry BA components of CB. **I** Venn diagram of PSC-related targets collected from four public databases. **J** Venn diagram identifying the overlapping targets between CB components and PSC. **K** Protein–protein interaction (PPI) network of the overlapping targets. **L**, **M** Significantly enriched GO terms **L** and KEGG pathways **M** for the overlapping targets

To elucidate the mechanism by which CB alleviates PSC, we performed liver transcriptome sequencing using samples from the DDC + Veh and DDC + CB (150 mg/kg) groups (n = 3 per group). Clustering analysis revealed clear separation between the DDC + Veh and DDC + CB groups (Fig. 2A). A total of 2030 differentially expressed genes (DEGs) were identified (|Log₂FC|> 1, FDR < 0.05), including 406 upregulated and 1624 downregulated genes (Fig. 2B). The top 100 DEGs are displayed in a heatmap (Fig. 2C). GO and KEGG enrichment analyses indicated that these DEGs were primarily associated with metabolic processes, transporter activity, PPAR signaling, steroid biosynthesis, fatty acid degradation, and bile secretion (Fig. 2D, E).

GSEA analysis of GO terms further revealed that biological processes, including “Bile acid biosynthetic process”, “Regulation of bile acid biosynthetic process”, “Bile acid metabolic process”, “Fatty acid beta-oxidation”, and “Fatty acid catabolic process” were significantly positively enriched after CB treatment (FDR < 0.01, Normalized ES > 2.10) (Fig. 2F). In GSEA analysis of KEGG pathways, “Primary bile acid biosynthesis”, “Bile secretion”, “PPAR signaling pathway”, and “Fatty acid degradation” were significantly positively enriched, while “Steroid biosynthesis” was negatively enriched after CB treatment (FDR < 0.064, Normalized ES > 1.40 or < − 1.70) (Fig. 2G).

To further investigate potential mechanisms, we performed a network pharmacology analysis based on the BA components of CB that entered the systemic circulation (Supplementary Table S1). SwissTargetPrediction analysis identified 435 potential targets for CB's bioactive components. The component-target relationships are shown in Fig. 2H. After cross-referencing in at least two databases, 1568 PSC-related targets were identified (Fig. 2I). A Venn diagram revealed 166 overlapping targets between the CB components and PSC, representing potential therapeutic targets of CB (Fig. 2J). Subsequent protein–protein interaction (PPI) network construction and hub gene analysis pinpointed FXR (NR1H4) and PPARα (PPARA) as core nodes in the network (Fig. 2K), which supports that the action of CB is closely related to the regulation of BA and lipid homeostasis. GO and KEGG analyses also confirmed the significant enrichment of these targets in lipid and BA metabolic pathways (Fig. 2L, M). These transcriptomic and network pharmacology findings strongly suggested that the therapeutic effect of CB is intimately linked to the reprogramming of BA and lipid metabolism. To validate this hypothesis and identify the key regulators, we next performed targeted metabolomics and molecular analyses.

### CB ameliorates DDC-induced PSC by modulating enterohepatic BA profiles

**Fig. 3:**
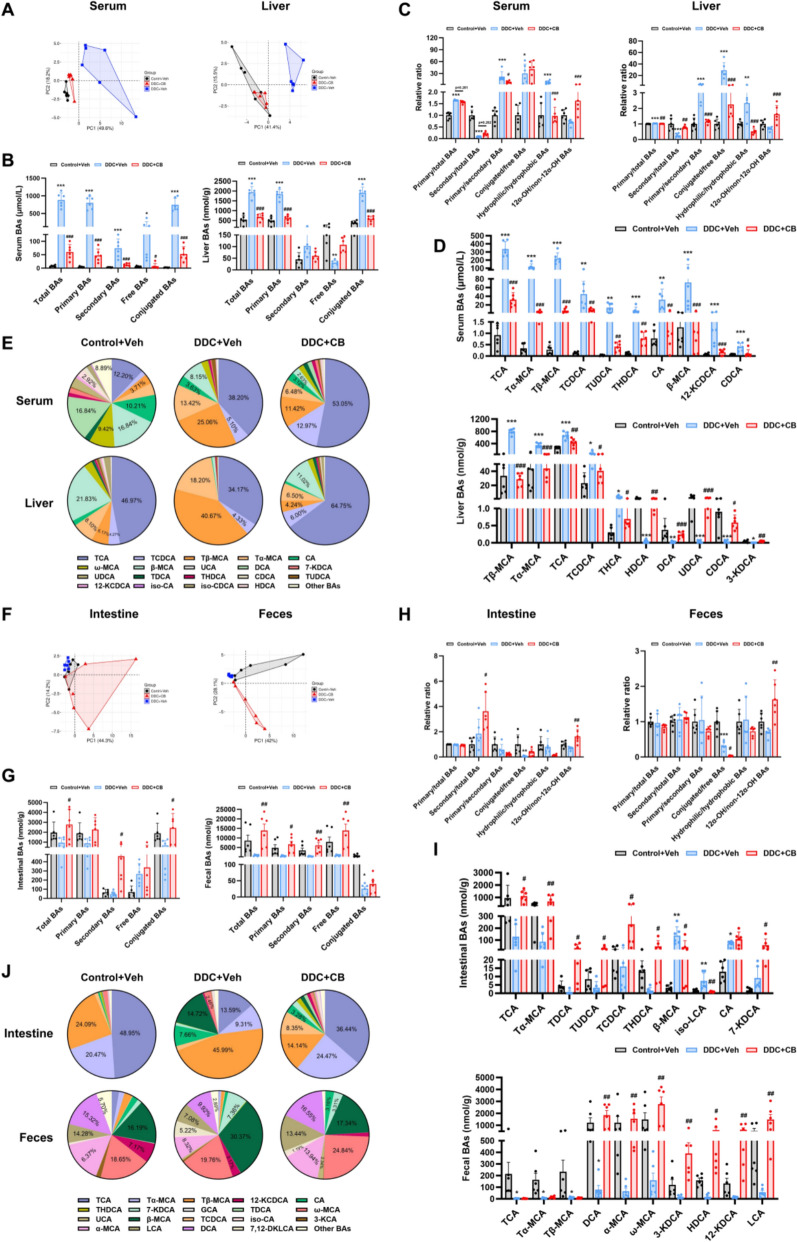
CB modulates BA profiles across the gut-liver axis in DDC-induced mice. **A** Principal component analysis (PCA) of the overall bile acid (BA) profiles in serum and liver. **B** Concentrations of total BAs, primary BAs, secondary BAs, free BAs, and conjugated BAs in serum and liver. **C** The ratios of various BA types in serum and liver. **D**, **E** Serum and hepatic levels of individual BAs, shown as absolute concentrations **D** and relative abundances **E**. **F** PCA of the overall BA profiles in the intestine and feces. **G** Concentrations of total BAs, primary BAs, secondary BAs, free BAs, and conjugated BAs in the intestine and feces. **H** The ratios of various BA types in the intestine and feces. **I**, **J** Levels of individual BAs in intestine and feces, shown as absolute concentrations **I** and relative abundances **J**. Data are presented as mean ± SEM (n = 6). ^*^p < 0.05, ^**^p < 0.01, ^***^p < 0.001 *vs*. Control + Veh; ^#^*p* < 0.05, ^##^*p* < 0.01, ^###^*p* < 0.001 *vs*. DDC + Veh. (Full name of the abbreviation for BA: see Supporting Table 2)

Given that dysregulated BA metabolism along the gut-liver axis is a key contributor to PSC [25], we employed targeted BA metabolomics to analyze the effects of CB on BA profiles in mouse serum, liver, intestine, and feces. Principal component analysis (PCA) of serum and liver samples showed a distinct separation of the DDC group from the Control and DDC + CB groups, with the latter two exhibiting highly similar or overlapping profiles (Fig. 3A), indicating a normalization of the BA profile by CB. Consistent with this, CB treatment significantly attenuated the DDC-induced elevations in total, primary, secondary, and conjugated BAs in both serum and liver (Fig. 3B). Notably, CB also restored the aberrant levels of free BAs, which were elevated in serum but reduced in liver by DDC challenge, to near-normal levels (Fig. 3B). Furthermore, CB administration induced a shift in key BA ratios towards a less cholestatic phenotype: it decreased the ratios of primary/total BAs, primary/secondary BAs, and hydrophilic/hydrophobic BAs in liver and serum, while increasing the ratios of secondary/total BAs and 12α-OH/non-12α-OH BAs (Fig. 3C). Analysis of individual BAs showed that CB mainly reduced the levels of conjugated BAs, such as TCA, Tα-MCA, Tβ-MCA, and TCDCA, in the serum and liver of DDC mice (Fig. 3D and Fig. S3A). CB also decreased serum free BAs such as CA, β-MCA, 12-KCDCA, and CDCA, while increasing hepatic free BAs, including HDCA, DCA, UDCA, CDCA, and 3-KDCA (Fig. 3D and Fig. S3A). Although the proportion of TCA remained elevated in the serum and liver after CB treatment, the relative abundances of other BAs trended toward those of the Control group (Fig. 3E).

In contrast to the serum and hepatic compartments, the BA profile in the intestine and feces presented a distinct response to CB. While PCA did not show clear separation among the experimental groups (Fig. 3F), quantitative analysis revealed that CB counteracted the DDC-induced reduction in total, primary, secondary, and conjugated BAs by increasing their levels in both intestine and feces (Fig. 3G). Critically, CB significantly increased the content of free BAs in feces and the secondary/total BA ratio in the intestine (Fig. 3G, H), suggesting enhanced microbiota-mediated BA deconjugation and secondary BA production, as well as increased fecal excretion. To further validate this, we performed 16S rRNA gene sequencing of fecal samples. The results showed that CB treatment markedly reversed the DDC-induced downregulation of key genera involved in deconjugation and secondary BA production, including *Akkermansia*, *Faecalibaculum*, *Lachnospiraceae_NK4A136_group*, and *Prevotellaceae_UCG-001*, which was consistent with the restored secondary BA profiles in the intestine and feces (Fig. S3C). Analysis of individual BAs confirmed that CB restored the content and proportion of multiple BAs in the intestine and feces to a pattern resembling that of the Control group (Fig. 3I, J, and Fig. S3B). Collectively, CB ameliorates PSC by reducing toxic BA burden in the liver and circulation, while promoting BA detoxification and elimination via the gut, thereby restoring enterohepatic BA homeostasis.

### CB normalizes the expression of key genes regulating BA metabolism and transport

**Fig. 4:**
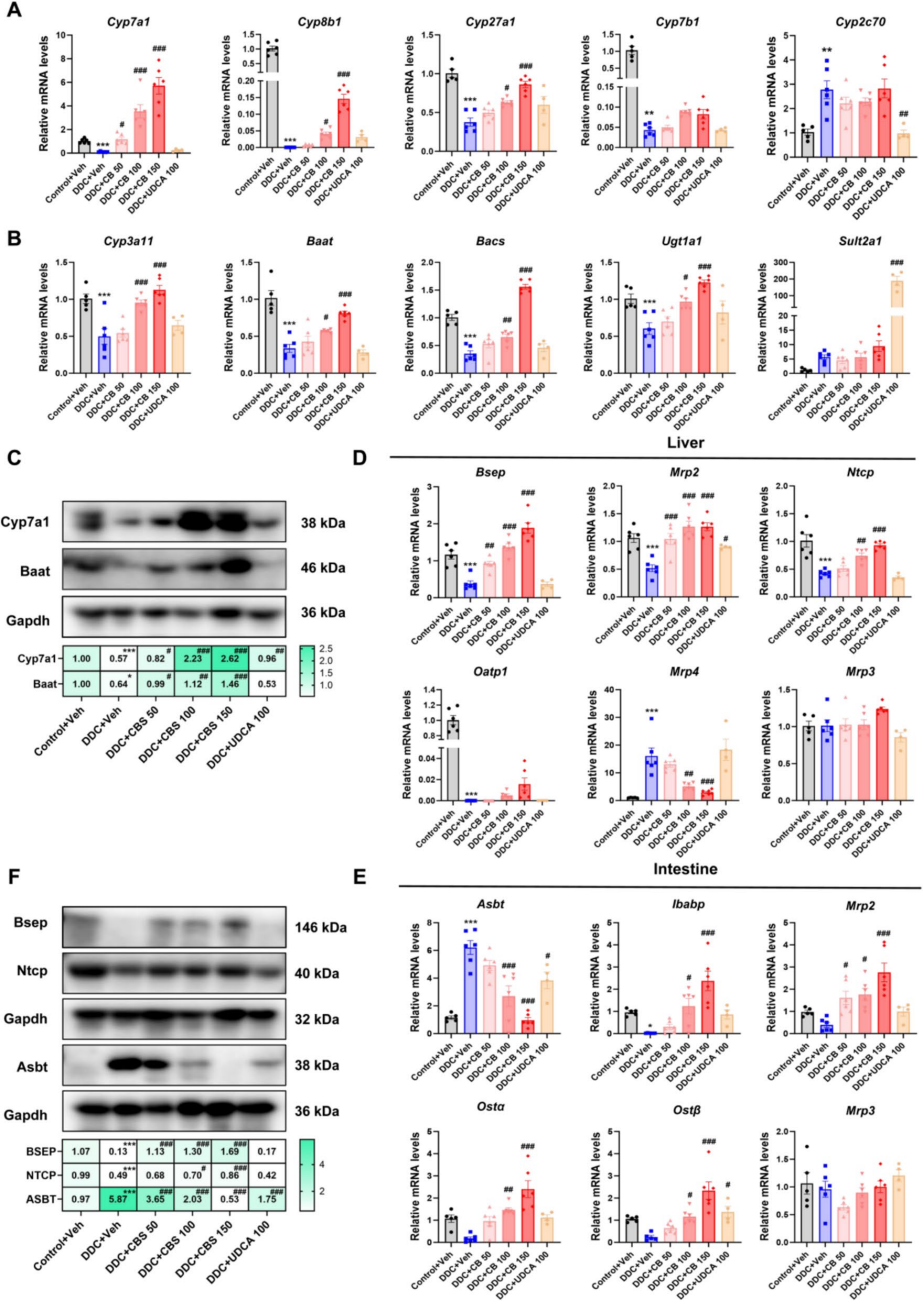
CB normalizes the expression of key genes regulating BA metabolism and transport in the liver and intestine. **A** Hepatic mRNA levels of genes involved in the classic (*Cyp7a1*, *Cyp8b1*) and alternative (*Cyp7b1*, *Cyp27a1*) BA synthesis pathways, as well as the murine-specific *Cyp2c70*, which generates muricholic acids. **B** Hepatic mRNA levels of genes responsible for BA phase I/II metabolism (*Cyp3a11*, *Ugt1a1*, *Sult2a1*, *Baat*, and *Bacs*). **C** Representative western blots and quantitative analysis of Cyp7a1 and Baat protein levels in the liver. **D** Hepatic mRNA levels of key transporters for BA uptake (*Ntcp*, *Oatp1*), canalicular export (*Bsep*, *Mrp2*), and basolateral efflux (*Mrp3*, *Mrp4*). **E** Intestinal mRNA levels of transporters for BA reabsorption, including the apical uptake transporter (*Asbt*), the intracellular carrier (*Ibabp*), and the basolateral efflux transporters (*Ostα*, *Ostβ*, *Mrp2*, *Mrp3*). **F** Representative western blots and quantitative analysis of Bsep and Ntcp protein levels in the liver, and Asbt protein levels in the ileum. All mRNA levels were determined by RT-qPCR and normalized to *Hprt1*. Protein levels were analyzed by western blotting and normalized to Gapdh. Data are presented as mean ± SEM (n = 4–6). ^*^p < 0.05, ^**^p < 0.01, ^***^p < 0.001 *vs*. Control + Veh; ^#^*p* < 0.05, ^##^*p* < 0.01, ^###^*p* < 0.001 *vs*. DDC + Veh

BA homeostasis in the gut-liver axis is tightly regulated by enzymes and transporters in both the intestine and liver [26]. Hepatic BA synthesis via the classical (Cyp7a1 and Cyp8b1) and alternative (Cyp7b1 and Cyp27a1) pathways was suppressed in DDC mice. CB treatment dose-dependently upregulated *Cyp7a1*, *Cyp8b1*, and *Cyp27a1* mRNA expression, but not *Cyp7b1* (Fig. 4A). UDCA had no significant effect on these synthetases. Hepatic Cyp2c70 (involved in muricholic acid synthesis) was increased by DDC and unaltered by CB, but reduced by UDCA (Fig. 4A). CB also modulated BA-metabolizing enzymes, dose-dependently increasing the DDC-suppressed mRNA expression of *Cyp3a11*, *Baat*, *Bacs*, and *Ugt1a1*, whereas UDCA showed no effect (Fig. 4B). Sult2a1 was increased by DDC and unaffected by CB but further elevated by UDCA (Fig. 4B). Western blot analysis confirmed corresponding changes in Cyp7a1 and Baat protein levels (Fig. 4C).

CB influenced hepatic BA transporters. Expression of the canalicular exporters *Bsep* and *Mrp2* was suppressed in DDC mice and significantly restored by CB (Fig. 4D). Similarly, the sinusoidal uptake transporters *Ntcp* and *Oatp1* were downregulated in the DDC group and upregulated by CB, with *Ntcp* returning to control levels (Fig. 4D). Among sinusoidal efflux transporters, *Mrp4* was elevated under DDC challenge, and significantly reduced by CB, whereas *Mrp3* remained unchanged (Fig. 4D). Protein expression of Bsep and Ntcp aligned with transcriptional changes (Fig. 4F). In the ileum, CB modulated key transporters involved in BA reabsorption and efflux. *Asbt*, which mediates BA uptake into enterocytes, was upregulated in DDC mice, and CB dose-dependently reduced its mRNA and protein expression (Fig. 4E, F). Conversely, CB upregulated *Ibabp*, *Ostα*, and *Ostβ*, all of which were downregulated in DDC animals (Fig. 4E). Intestinal *Mrp3* expression remained unaltered across groups (Fig. 4E).

### CB ameliorates disordered lipid metabolism across the gut-liver axis in DDC-induced PSC mice

**Fig. 5:**
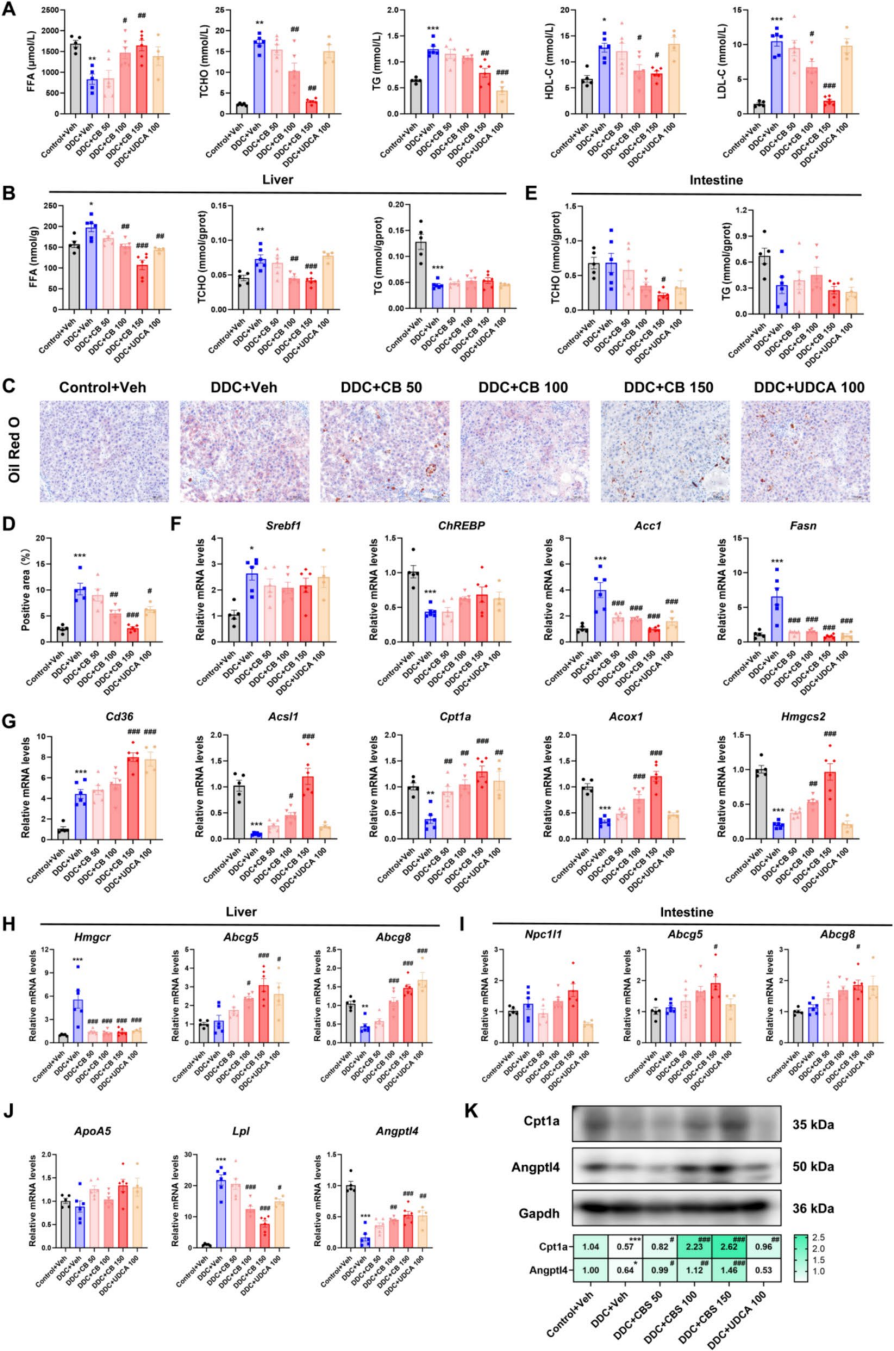
CB ameliorates disordered lipid metabolism across the gut-liver axis in DDC-induced PSC mice. **A** Serum levels of free fatty acid (FFA), total cholesterol (TCHO), triglycerides (TG), high-density lipoprotein cholesterol (HDLC), and low-density lipoprotein cholesterol (LDLC). **B** Hepatic levels of FFA, TCHO, and TG. **C** Representative images and **D** quantification of hepatic lipid deposition as assessed by Oil Red O staining. **E** Ileal levels of TCHO and TG. **F** Hepatic mRNA levels of lipogenic genes (*Srebf1*, *ChREBP*, *Acc1*, *Fasn*). **G** Hepatic mRNA levels of genes involved in fatty acid β-oxidation (*Cd36, Acsl1*, *Cpt1a*, *Acox1*, *Hmgcs2*). **H** Hepatic mRNA levels of genes regulating cholesterol synthesis (*Hmgcr*) and efflux (*Abcg5/8*). **I** Ileal mRNA levels of genes involved in cholesterol absorption (*Npc1l1*) and trans-intestinal excretion (*Abcg5/8*). **J** Hepatic mRNA levels of key regulators of plasma TG clearance (*ApoA5*, *Lpl*, *Angptl4*). **K** Representative western blots and quantification of Cpt1a and Angptl4 protein levels in liver. All mRNA levels were determined by RT-qPCR and normalized to *Hprt1*. Protein levels were analyzed by western blotting and normalized to Gapdh. Data are presented as mean ± SEM (n = 4–6). ^*^p < 0.05, ^**^p < 0.01, ^***^p < 0.001 *vs*. Control + Veh; ^#^*p* < 0.05, ^##^*p* < 0.01, ^###^*p* < 0.001 *vs*. DDC + Veh

Impaired BA excretion disrupts lipid homeostasis and aggravates oxidative stress [27, 28]. In the serum of DDC-fed mice, levels of TCHO, TG, HDLC, and LDLC were significantly elevated, while FFAs were reduced. CB treatment dose-dependently reversed these alterations, effectively lowering TCHO, TG, HDLC, and LDLC, while restoring FFA content (Fig. 5A). In liver tissue, FFA and TCHO levels were elevated in the DDC group, whereas TG was reduced, and CB effectively lowered hepatic FFA and TCHO but did not significantly alter TG content (Fig. 5B). Oil Red O staining revealed substantial lipid droplet accumulation in DDC mouse livers, which was markedly attenuated by CB, with high-dose CB showing superior efficacy to UDCA (Fig. 5C, D). Additionally, CB also reduced intestinal TCHO without significantly affecting TG levels (Fig. 5E), indicating a systemic improvement in lipid metabolism spanning the gut-liver axis.

We next investigated the molecular mechanisms underlying these improvements. In the liver, CB significantly downregulated DDC-induced mRNA expression of lipogenic genes *Acc1* and *Fasn*, without affecting key transcription factors *Srebf1* and *ChREBP* (Fig. 5F). Importantly, CB dose-dependently enhanced the expression of genes critical for fatty acid β-oxidation, including *Cd36*, *Acsl1*, *Cpt1a*, *Acox1*, and *Hmgcs2* (Fig. 5G), indicating a promoted shift toward fatty acid catabolism. With regard to cholesterol metabolism, CB suppressed the DDC-induced upregulation of hepatic *Hmgcr*, a key rate-limiting enzyme in cholesterol synthesis (Fig. 5G). CB also promoted the expression of cholesterol efflux transporters *Abcg5* and *Abcg8* in liver and intestine, facilitating cholesterol excretion into bile and the intestinal lumen, which likely contributed to the observed reduction in hepatic and intestinal TCHO content (Fig. 5H, I). The expression of cholesterol uptake transporter *Npc1l1* remained unchanged (Fig. 5I). In triglyceride metabolism, CB suppressed hepatic *LpL* and elevated *Angptl4* mRNA levels (Fig. 5J), changes predicted to attenuate TG hydrolysis into FFA and monoglycerides. Hepatic *ApoA5* expression remained unaltered (Fig. 5J). Western blot analysis confirmed increased protein levels of Cpt1a (promoting fatty acid β-oxidation) and Angptl4 (inhibiting TG hydrolysis) upon CB treatment (Fig. 5K), supporting its role in restoring lipid metabolic balance across the gut-liver axis.

### CB modulates BA and lipid homeostasis through upregulating intestinal and hepatic FXR/PPARα through the SIRT1-PGC-1α axis

**Fig. 6:**
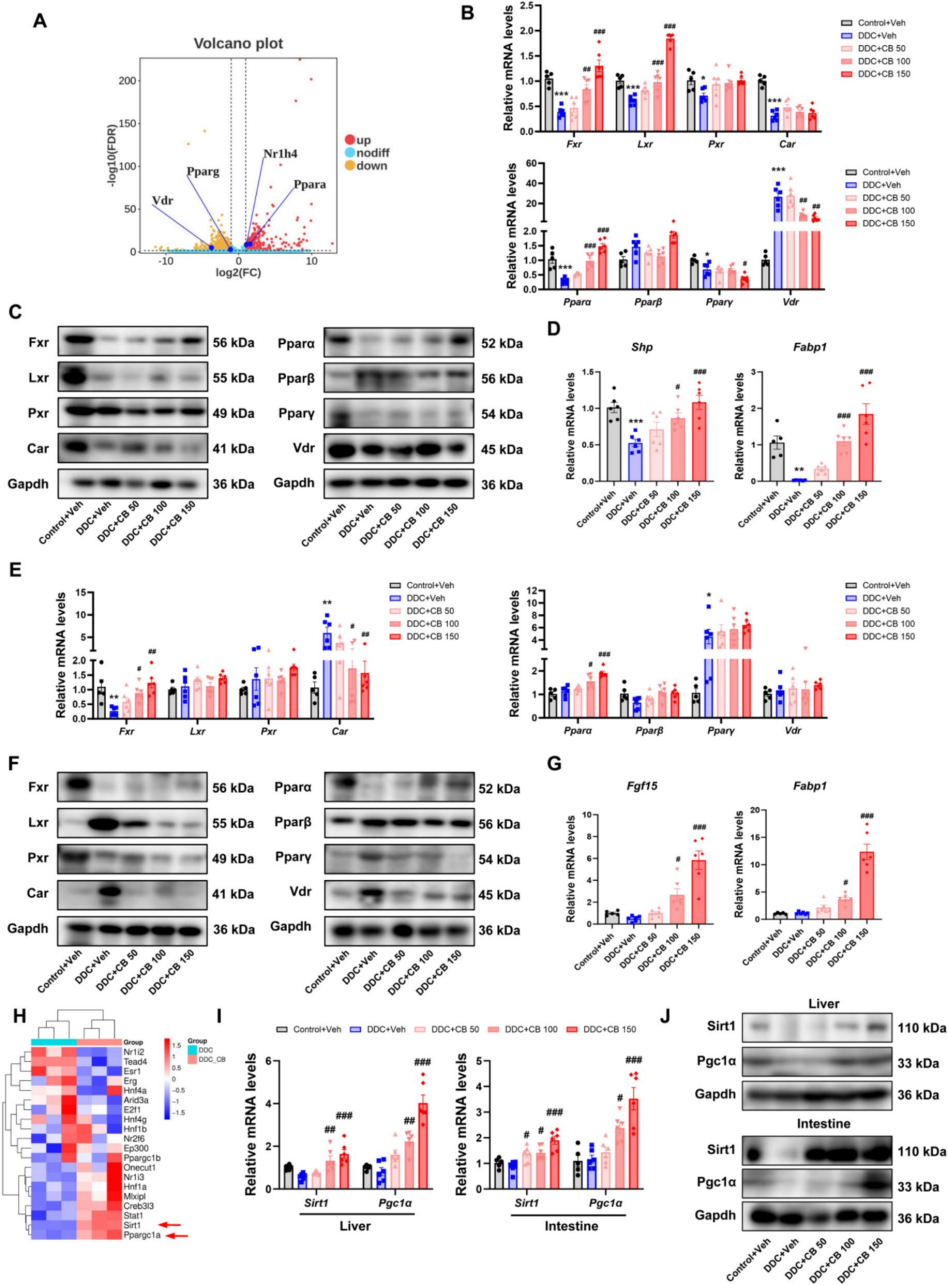
CB upregulates intestinal and hepatic FXR and PPARα via the SIRT1-PGC-1α axis. **A** Volcano plot of liver transcriptome data highlighting upregulation of *Fxr* and *Pparα* by CB. **B** Hepatic mRNA levels of nuclear receptors. **C** Representative western blots of hepatic nuclear receptor proteins. **D** Hepatic mRNA levels of *Shp* (FXR target) and *Fabp1* (PPARα target). **E** Ileum mRNA levels and **F** representative western blots of nuclear receptors. **G** Ileum mRNA levels of *Fgf15* (FXR target) and *Fabp1* (PPARα target). **H** Screening of upstream regulators from transcriptome data identified SIRT1 and PGC-1α as top candidates. **I** mRNA and **J** protein levels of SIRT1 and PGC-1α in liver and intestine. All mRNA levels were determined by RT-qPCR and normalized to *Hprt1*. Protein levels were analyzed by western blotting and normalized to Gapdh. Data are presented as mean ± SEM (n = 4–6). ^*^p < 0.05, ^**^p < 0.01, ^***^p < 0.001 *vs*. Control + Veh; ^#^*p* < 0.05, ^##^*p* < 0.01, ^###^*p* < 0.001 *vs*. DDC + Veh

Having established that CB restores BA and lipid homeostasis, we sought to identify the master transcriptional regulators mediating these benefits. Given the central role of nuclear receptors (NRs) in coordinating metabolism, we analyzed their expression profiles. A volcano plot of transcriptome data indicated that CB significantly upregulated *Fxr (Nr1h4)* and *Pparα (Ppara)*, downregulated *Pparγ (Pparg)* and *Vdr* (Fig. 6A). This finding prompted us to systematically validate the expression of key NRs in liver tissue. RT‑qPCR analysis confirmed that DDC challenge decreased mRNA levels of *Fxr*, *Lxr*, *Pxr*, *Car*, *Pparα*, and *Pparγ*, increased *Vdr*, and left *Pparβ/δ* unchanged*.* CB treatment dose‑dependently upregulated *Fxr*, *Pparα*, and *Lxr* mRNA, suppressed *Vdr*, and did not affect other NRs (Fig. 6B). At the protein level, DDC reduced FXR, LXR, PXR, CAR, PPARα, and PPARγ, elevated PPARβ/δ, and slightly decreased VDR. Remarkably, CB selectively and dose‑dependently increased only FXR and PPARα protein; other NRs remained unchanged or, in the case of CAR, were further downregulated (Fig. 6C and Fig. S4A). Consistently, CB elevated the expression of downstream targets *Shp* (FXR target) and *Fabp1* (PPARα target) (Fig. 6D), demonstrating selective activation of hepatic FXR and PPARα signaling.

We next examined whether CB exerted similar regulatory effects on NRs in the intestine, a key compartment for BA and lipid metabolism in the gut-liver axis. In DDC mice, intestinal mRNA levels of *Fxr* and *Pparα* were decreased, while *Car* and *Pparγ* were increased; *Lxr*, *Pxr*, *Pparβ/δ*, and *Vdr* remained unchanged. CB dose‑dependently increased *Fxr* and *Pparα* mRNA and reduced *Car* (Fig. 6E). Protein analysis confirmed decreased FXR, PPARα, and PXR and increased LXR, CAR, and PPARγ in DDC mice, while CB selectively upregulated FXR and PPARα protein (Fig. 6F and Fig. S4B). Correspondingly, CB increased the expression of intestinal *Fgf15* (Fxr target) and *Fabp1* (Pparα target) (Fig. 6G). Thus, CB concurrently upregulates and activates FXR and PPARα in both liver and intestine.

To identify the upstream mechanism responsible for the coordinated upregulation of FXR and PPARα, we screened potential transcriptional regulators using the ChEA3 and TRRUST databases or from the literature. Liver transcriptome data highlighted *Sirt1* as one of the most significantly upregulated candidates (Fig. 6H). Given that PGC‑1α serves as a critical co‑activator for both FXR and PPARα [29, 30], and SIRT1 deacetylates and activates PGC‑1α [31, 32], we hypothesized that CB acts through the SIRT1-PGC-1α axis. Indeed, CB treatment increased both mRNA and protein levels of SIRT1 and PGC‑1α in the liver and intestine (Fig. 6I, J, and Fig. S4C-D). This supports the role of the SIRT1/PGC‑1α pathway in mediating CB‑induced upregulation of FXR and PPARα across the gut-liver axis.

### *In vitro validation of CB-mediated upregulation of FXR/PPARα through the SIRT1-PGC-1α axis*

**Fig. 7:**
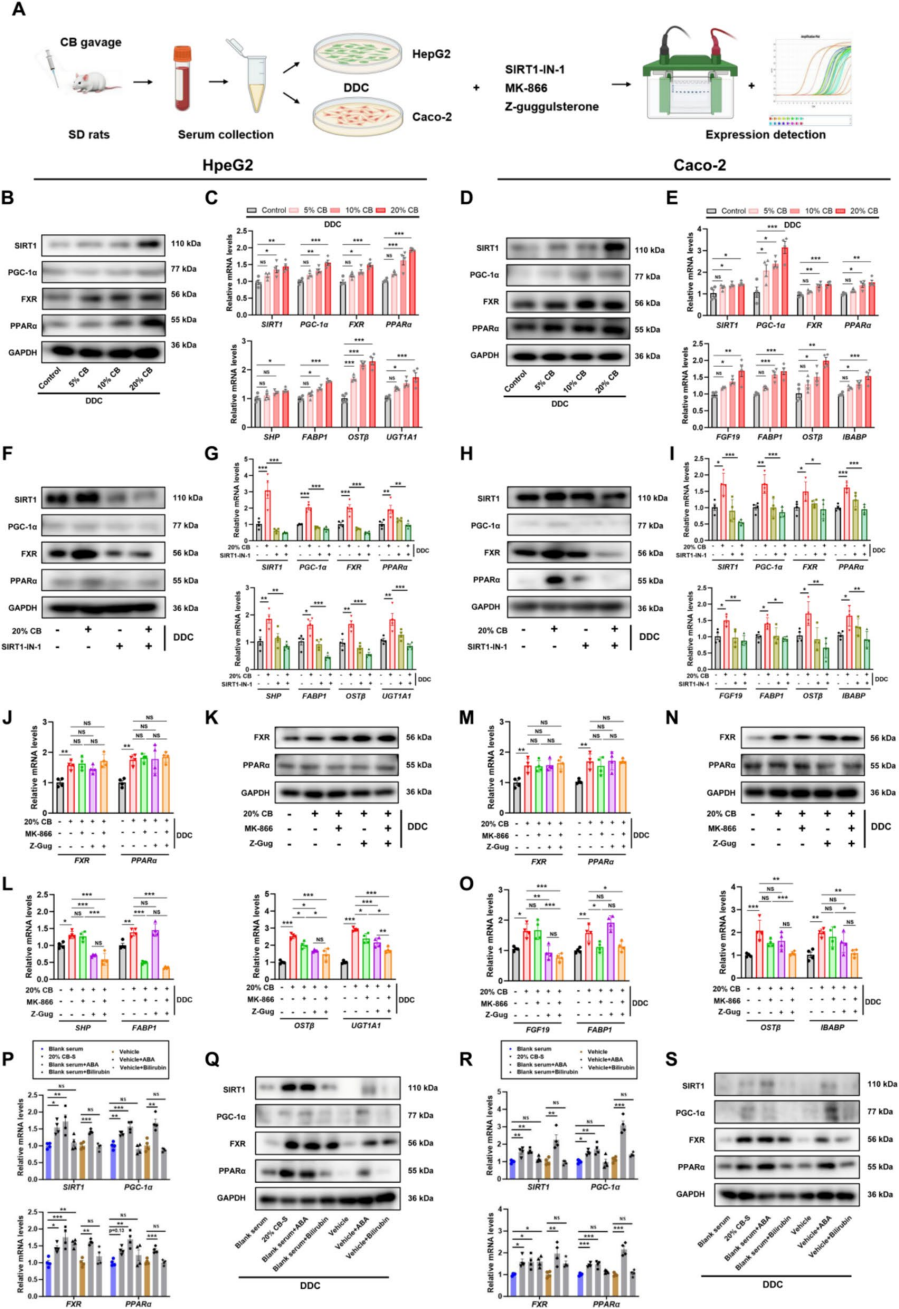
*In vitro* validation of the SIRT1-PGC-1α-FXR/PPARα axis as the mechanistic core for CB's action. **A** Schematic overview of the *in vitro* experimental design. **B**–**E** HepG2 and Caco-2 cells were treated with DDC and increasing concentrations of CB-containing serum (CB-S). **B**, **D** Western blots of SIRT1, PGC-1α, FXR, and PPARα in HepG2 **B** and Caco-2 **D** cells. **C**, **E** RT-qPCR analysis of indicated genes in HepG2 **C** and Caco-2 **E** cells. **F**–**I** Cells pre-treated with SIRT1 inhibitor SIRT1-IN-1 before DDC + 20% CB-S. **F**, **H** Western blots of SIRT1, PGC-1α, FXR, and PPARα in HepG2 **F** and Caco-2 **H** cells. (G, I) RT-qPCR analysis of indicated genes in HepG2 **G** and Caco-2 **I** cells. **J**–**O** Cells treated with DDC + 20% CB-S, and FXR antagonist (Z-guggulsterone, Z-gug) and/or PPARα antagonist (MK-886). (J, M) RT-qPCR analysis of *FXR* and *PPARα* mRNA levels in HepG2 **J** and Caco-2 M cells. **K**, **N** Western blots of FXR and PPARα in HepG2 **K** and Caco-2 **N** cells. **L**, **O** RT-qPCR analysis of indicated genes in HepG2 **L** and Caco-2 **O** cells. **P**–**S** DDC-injured HepG2 and Caco-2 cells were treated with ABA or bilirubin in the presence or absence of serum. **P**, **R** RT-qPCR analysis of indicated genes in HepG2 **P** and Caco-2 **R** cells. **Q**, **S** Western blots of SIRT1, PGC-1α, FXR, and PPARα in HepG2 **Q** and Caco-2 **S** cells. All mRNA levels were determined by RT-qPCR and normalized to *GAPDH*. Protein levels were analyzed by western blotting and normalized to GAPDH. Data are presented as mean ± SEM (n = 3–4). *p < 0.05, **p < 0.01, ***p < 0.001

To establish a direct causal relationship and validate the central role of the SIRT1-PGC-1α-FXR/PPARα axis identified *in vivo*, we performed a series of in vitro pharmacological interventions using human hepatic (HepG2) and intestinal (Caco-2) cell lines (Fig. 7A). Consistent with our *in vivo* findings, treatment with CB-S (5, 10, 20%) in DDC-injured HepG2 and Caco-2 cells resulted in a dose-dependent upregulation of the key components of the identified axis. Both mRNA and protein levels of SIRT1, its downstream coactivator PGC-1α, and the NRs FXR and PPARα were significantly increased by CB-S treatment compared to cells treated with Con-S (Fig. 7B, D; Fig. S5C-D). Consequently, CB-S upregulated the mRNA expression of FXR target genes (*SHP*, *FGF19*), PPARα target gene (*FABP1*), and genes known to be regulated by both receptors (*OSTB*, *UGT1A1*, *IBABP*) in both cell lines (Fig. 7C, E).

To determine the upstream regulatory role of SIRT1, we employed the specific SIRT1 inhibitor SIRT1-IN-1. Pre-treatment with SIRT1-IN-1 significantly attenuated the CB-S-induced upregulation of SIRT1, PGC-1α, FXR, and PPARα at both mRNA and protein levels (Fig. 7F–I and Fig. S5C-D) and reversed the induction of their downstream targets (Fig. 7F–I). SIRT1-IN-1 alone had no significant effect on these parameters, confirming its specific antagonism of CB-S-induced signaling.

To verify FXR and PPARα as key downstream effectors, we used their respective pharmacological antagonists, Z-guggulsterone (FXR) and MK-886 (PPARα). As expected, neither antagonist alone nor in combination altered the CB-S-induced mRNA or protein expression of FXR or PPARα themselves (Fig. 7J–O, and Fig. S5C-D). However, they significantly blunted the induction of downstream genes. In both HepG2 and Caco-2 cells, Z-guggulsterone specifically inhibited the expression of FXR target genes (*SHP* and *FGF19*), while MK-886 specifically suppressed the PPARα target gene (*FABP1*) (Fig. 7L, O). For common target genes (*OSTB*, *UGT1A1*, *IBABP*), each antagonist significantly reduced their CB-S-induced expression, with the combined use of both antagonists producing the strongest inhibitory effect (Fig. 7L, O).

To dissect the role of BAs in CB-S and exclude non-specific interference, an ABA mixture was formulated based on the concentrations of the ten most abundant BAs detected in 20% CB-S. An equimolar bilirubin solution was prepared in parallel. In DDC-injured HepG2 and Caco-2 cells, ABA or bilirubin was either supplemented into Con-S or applied directly in serum-free medium. ABA treatment significantly upregulated SIRT1, PGC‑1α, FXR, PPARα, and their downstream target genes in both cell lines, closely recapitulating the effects of CB-S (Fig. 7P–S and Fig. S5C-F). In contrast, bilirubin only mildly increased FXR and its target genes, with no significant effect on SIRT1, PGC‑1α, or PPARα (Fig. 7P–S and Fig. S5C-F). These results indicate that the prototype BA constituents in CB are the primary substances responsible for activating the SIRT1‑PGC‑1α‑FXR/PPARα axis and mediating the subsequent metabolic protective effects.

### CB components directly bind to and activate FXR and PPARα

Molecular docking analysis revealed that major BA components abundant in CB and capable of entering the bloodstream could stably dock into the ligand-binding pockets of both FXR and PPARα (Fig. S6A). The calculated binding energies for these compounds ranged from − 6.57 to − 10.05 kcal/mol, suggesting strong potential for direct interaction (Fig. S6A). Importantly, CDCA, CA, and DCA are well-established endogenous ligands for FXR [33], and CDCA, CA, and HDCA have also been reported as PPARα agonists [34, 35]. Representative conformational structures from the molecular docking of CDCA with PPARα and FXR are presented in Fig. S6B. *In vitro* studies using HepG2 and Caco-2 cells demonstrated that CDCA, CA, and DCA significantly upregulated the mRNA expression of FXR target genes (*SHP* or *FGF19*), while CDCA, CA, and HDCA markedly increased the mRNA expression of the PPARα target *FABP1*, without affecting *FXR* and *PPARα* mRNA levels themselves (Fig. S6C). These results indicate that CB not only upregulates the expression of FXR and PPARα, but also provides direct pharmacological agonists for these two NRs, leading to their functional activation.

## Discussion

**Fig. 8:**
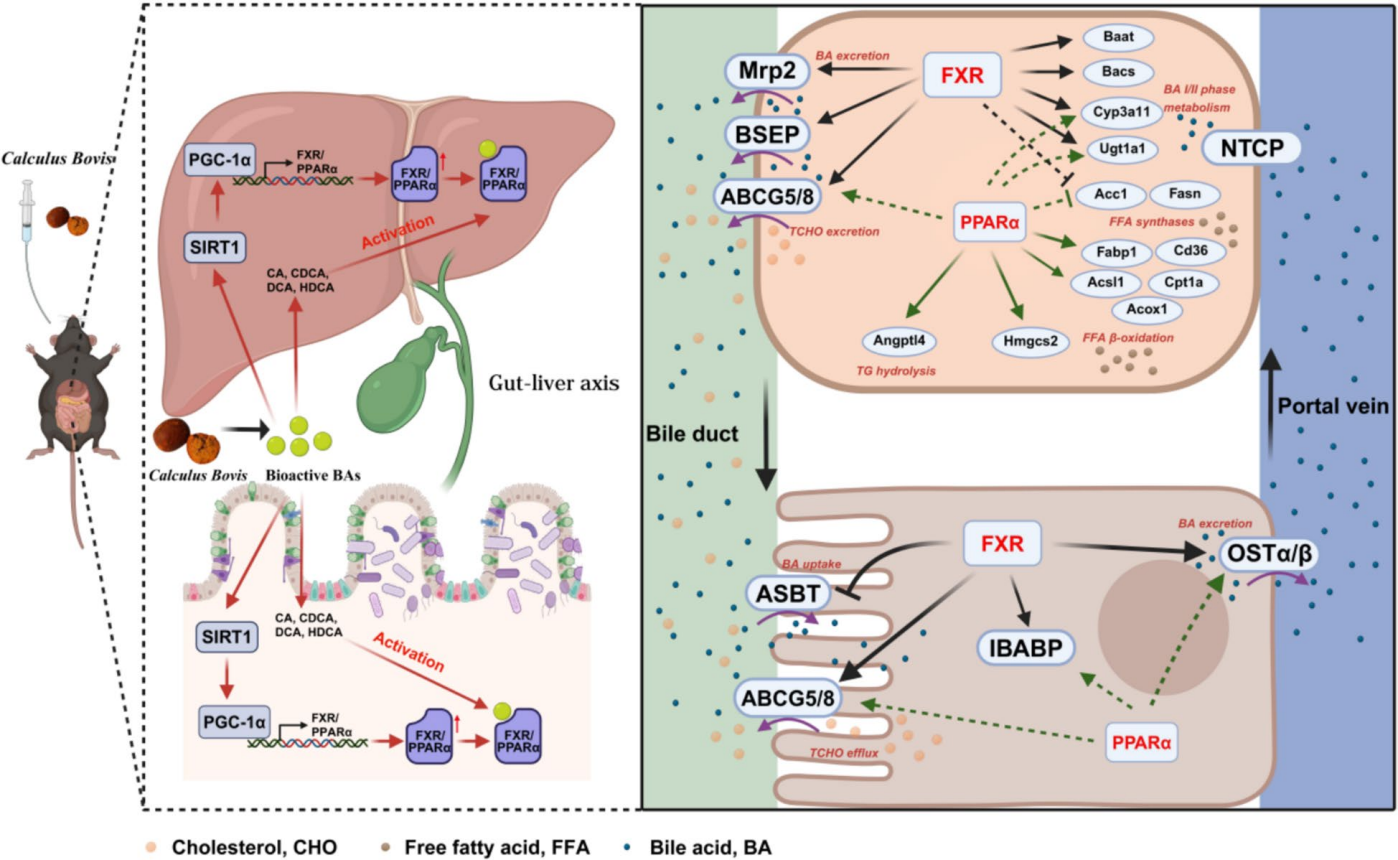
Schematic diagram illustrating the proposed mechanism by which CB ameliorates PSC. CB and its bioactive BA components alleviate PSC by restoring bile acid and lipid homeostasis via the gut-liver axis through a dual mechanism: (1) transcriptional upregulation of FXR and PPARα in hepatocytes and enterocytes through activation of the SIRT1-PGC-1α axis; and (2) direct ligand-dependent activation of FXR and PPARα in these cells. This concerted action enhances the transcription of genes involved in bile acid detoxification, transport, and fatty acid β-oxidation, thereby reducing hepatic injury, fibrosis, and intestinal barrier damage

PSC remains a formidable clinical challenge as a progressive cholestatic liver disease without FDA-approved pharmacotherapy. Liver transplantation, while curative, is limited by donor availability and risk of disease recurrence [4]. This underscores the urgent need for novel therapeutic strategies targeting the core pathological drivers of PSC. Our study provides compelling preclinical evidence that CB, a traditional medicine derived from animal gallstones, confers significant therapeutic benefits in an experimental model of PSC. The core efficacy of CB lies in the comprehensive restoration of the dual homeostasis of BA and lipid metabolism within the gut-liver axis. Mechanistically, we have revealed a dual-pronged mechanism whereby CB and its bioactive BA components: (1) transcriptionally upregulate FXR/PPARα expression via activating the SIRT1-PGC-1α axis; and (2) directly serve as endogenous ligands to agonize the protein function of FXR/PPARα. This synergistic effect ultimately enhances the transcriptional program of BA detoxification, efflux, and fatty acid β-oxidation (Fig. 8).

Metabolic disturbances, particularly the dysregulation of BA and lipid homeostasis, represent core pathological features of PSC and form a vicious cycle that drives disease progression. Patients with PSC exhibit a characteristic BA profile marked by increased serum levels of total, conjugated, and primary BAs, alongside a reduced fecal BA excretion, reflecting impaired enterohepatic circulation and detoxification [36–38]. This dysregulation of BA metabolism is intrinsically linked to profound lipid metabolic disorders, frequently manifesting as hyperlipidemia, altered serum lipoprotein profiles, and impaired mitochondrial fatty acid β-oxidation, which collectively exacerbate oxidative stress and hepatocellular injury [39–41]. Notably, elevated serum cholesterol has been correlated with adverse outcomes in cholestasis, and modulation of lipid metabolism can ameliorate liver injury in experimental models, highlighting its therapeutic relevance [27, 42]. In our study, the DDC-induced mouse model recapitulated these clinical hallmarks, displaying aberrant BA profiles and significant lipid disturbances. CB treatment effectively reversed these interconnected metabolic pathologies. It normalized the BA profile across the gut-liver axis by reducing the hepatic and systemic burden of toxic BAs while promoting their intestinal detoxification and fecal excretion. Concurrently, CB ameliorated dyslipidemia, lowered hepatic and serum cholesterol and fatty acid levels, and reduced hepatic lipid accumulation. These coordinated effects underscore CB's capacity to simultaneously rectify the intertwined BA and lipid metabolic derangements that are central to PSC pathogenesis, suggesting a systems-level therapeutic intervention rather than isolated pathway modulation.

Integrated multi-omics and pathway synergistic validation strategies have been effectively applied in recent studies to elucidate the mechanisms of action of natural products [43]. Through integrated transcriptomic and network pharmacology analyses, we identified FXR and PPARα as core targets of CB. FXR and PPARα, highly expressed in both the liver and ileum, are master regulators of BA and lipid homeostasis, respectively, in the gut-liver axis [26, 44, 45]. Subsequent validation confirmed that CB selectively upregulates FXR and PPARα expression and activates their downstream target genes in both the liver and intestine. Hepatic FXR activation enhanced the expression of detoxifying enzymes (Cyp3a11, Baat, Bacs, and Ugt1a1) and efflux transporters (Bsep and Mrp2), while intestinal FXR activation suppressed the reuptake transporter Asbt and promoted basolateral efflux (Ibabp, Ostα, and Ostβ). Concurrently, hepatic PPARα activation promoted the expression of genes involved in fatty acid transport (Cd36), activation (Acsl1), β-oxidation (Cpt1a, Acox1), and ketoacidogenesis (Hmgcs2), thereby enhancing lipid catabolism. The upregulation of Angptl4, a PPARα target, likely contributed to reduced triglyceride hydrolysis [46].

Crucially, FXR and PPARα exhibit cross-regulatory roles in BA and lipid metabolism [47, 48]. FXR directly promotes the transcription of cholesterol efflux transporters ABCG5/8, while PPARα can indirectly enhance their expression [49, 50]. Although neither FXR nor PPARα directly targets fatty acid synthases ACC1 and FASN, both can mediate their indirect suppression. In this study, CB was shown to upregulate Abcg5/Abcg8 in both liver and ileum and inhibit Acc1 and Fasn in the liver, thereby promoting cholesterol efflux into bile and intestine and reducing de novo fatty acid synthesis in the liver. In BA metabolism, PPARα can exert direct or indirect regulatory effects on multiple genes, such as inhibiting CYP7A1 and CYP27A1, and upregulating CYP3A4, UGT1A1, SULT2A1, as well as transporters ASBT, IBABP, and OSTβ [47, 51]. *In vitro* experiments further confirmed that FXR and PPARα are key mediators of CB's downstream effects. Dual antagonism of both receptors further diminished CB's upregulatory effect on the common target genes UGT1A1, IBABP, and OSTβ.

Notably, CB activated both intestinal and hepatic FXR. While FXR activation typically suppresses the expression of the classical (Cyp7a1, Cyp8b1) and alternative (Cyp27a1) BA synthesis pathways [26], these synthetic enzymes were upregulated in CB-treated mice. This likely reflects compensatory feedback regulation following cholestasis amelioration, a phenomenon similarly reported for other FXR agonists [52]. Consistent with this, hepatic levels of the primary BAs CA and CDCA were reduced in DDC-induced mice but restored by CB treatment, corroborating the recovery of hepatic BA synthesis. Conversely, serum CA and CDCA levels were markedly elevated in DDC mice and significantly lowered by CB, directly demonstrating the alleviation of cholestatic accumulation. Mechanistically, this is attributed to: (1) upregulation of the canalicular efflux transporters Bsep and Mrp2, restoring hepatic BA excretion; (2) suppression of the intestinal reuptake transporter Asbt, reducing enterohepatic recycling and promoting fecal elimination; and (3) induction of the metabolizing enzymes UGT1A1 and SULT2A1, facilitating the conjugation and clearance of CA and CDCA from the circulation.

A key finding of this study is the elucidation of the upstream pathway leading to FXR/PPARα upregulation. We identified that CB activates the SIRT1-PGC-1α axis, and that the absorbed BA components of CB are the core substances mediating this upregulation. SIRT1, an NAD + -dependent deacetylase, is involved in regulating various physiological processes, including growth, development, aging, and metabolism [53]. It directly or indirectly modulates several key NRs, including CAR, FXR, PXR, PPARα, and HNF4α [54, 55]. Under pathological conditions such as cholestasis and hepatic steatosis, SIRT1 expression is typically downregulated [56, 57]. In contrast, SIRT1 overexpression or treatment with small-molecule activators has been shown to alleviate diet-induced steatosis and cholestatic injury in mice [56, 58]. Furthermore, SIRT1 promotes FXR transcriptional activity by directly deacetylating FXR at Lys217, facilitating its binding to FXRE promoter sequences [56]. SIRT1 also deacetylates and activates PGC-1α, a key transcriptional coactivator [59]. PGC-1α, in turn, enhances both FXR and PPARα mRNA levels via HNF4α activation and directly interacts with their DNA-binding domains to potentiate the transcription of downstream target genes [29, 30]. Our *in vitro* experiments confirmed that a SIRT1 inhibitor blocked CB-induced upregulation of this entire axis, positioning SIRT1 as a critical upstream mediator and a potential therapeutic target in PSC.

Previous reports indicate that the main components of CB, bilirubinand BAs can both upregulate SIRT1 expression [56, 60, 61]. Through component verification experiments using an ABA mixture and bilirubin, we further clarified that the effects observed with CB-S are attributable to its BA components, rather than host secondary metabolites or nonspecific serum factors, and we ruled out a major contribution of bilirubin to the SIRT1-PGC-1α axis. The mechanisms by which ABA upregulates the SIRT1-PGC-1α axis may involve: (1) BA-mediated activation of the AMPK-SIRT1 signaling axis via the TGR5 receptor [62]; and (2) BA-dependent post-transcriptional regulation of SIRT1, such as modulation of miR-34a expression [56]. The precise mechanisms warrant further in-depth investigation.

More importantly, the mechanism of CB is not limited to transcriptional upregulation of FXR/PPARα. Molecular docking and *in vitro* assays indicated that its inherent BA components, such as CA, CDCA, HDCA, and DCA, can directly bind to the ligand-binding pockets of FXR and PPARα and activate their protein function. This forms a unique synergistic strategy of “transcriptional sensitization + direct ligand activation”: CB and its bioactive BA components concurrently upregulate the expression of FXR/PPARα via the SIRT1-PGC-1α axis and provide endogenous ligands to directly activate these receptors. This mode not only recapitulates the clinical strategy of combined use of FXR and PPARα agonists, but also avoids the limitations of the limited efficacy of single-target drugs and additive adverse effects of combination therapy [47, 63, 64], which is highly suitable for the treatment of complex and multifactorial diseases such as PSC.

For clinical translation, the 50–150 mg/kg/day doses of CB in mice correspond to a human equivalent dose (HED) of 4.06–12.17 mg/kg/day for a 60 kg adult. The lower end of this HED range fully aligns with the clinical dosage of natural CB in the *Chinese Pharmacopoeia (2025 Edition)* (2.5–5.83 mg/kg/day), and no obvious toxicity was observed in mice even at the highest dose, confirming a good safety window. For economic feasibility, despite the limited resources and high cost of natural CB, *in vitro* cultured CB, officially included in the *Chinese Pharmacopoeia (2025 Edition)* with equivalent clinical substitution eligibility and consistent pharmacodynamic properties to natural CB, has achieved large-scale production [17, 18], greatly reducing costs and improving clinical accessibility for potential PSC treatment.

This study has several limitations. First, although *in vitro* pharmacological inhibition strongly supports the central role of the SIRT1-PGC-1α axis, in vivo causal validation using tissue-specific *Sirt1* knockout mice or SIRT1 inhibitors in the DDC model is still needed to confirm the indispensable role of this pathway. Second, which specific BA monomers in CB upregulate SIRT1 and through what molecular mechanisms remain unclear, warranting further structure–activity relationship and target identification studies. Third, fecal BA profile alterations are tightly linked to gut microbiota remodeling. Our preliminary 16S rRNA sequencing confirmed CB restored key secondary BA-producing genera, aligning with secondary BA homeostasis recovery. However, the causal role of gut microbiota in CB’s anti-PSC effect remains unvalidated, with detailed mechanisms under investigation in our follow-up study. Finally, the DDC diet model used in this study primarily simulates the pathological processes of bile duct injury and cholestasis but does not fully recapitulate all features of human PSC. Future validation in additional PSC animal models (e.g., Mdr2^−/−^ knockout mice) is needed to comprehensively evaluate its clinical translational potential.

In conclusion, this study demonstrates that CB attenuates experimental PSC by orchestrating a repair of BA and lipid homeostasis via the gut-liver axis. Its efficacy is driven by a novel dual mechanism of activating the SIRT1-PGC-1α pathway to upregulate FXR/PPARα expression, coupled with direct receptor agonism. These findings not only position CB as a promising multi-target candidate for PSC treatment, but also provide a novel framework for therapeutically modulating metabolic crosstalk within the gut-liver axis.

## Supplementary Information

Supplementary Material 1

## Data Availability

The datasets used or analyzed throughout this study are available from the corresponding author upon reasonable request.

## Abbreviations

PSC: Primary sclerosing cholangitis
FDA: The U.S. Food and Drug Administration
UDCA: Ursodeoxycholic acid
Bas: Bile acids
FXR: Farnesoid X receptor
PPARα: Peroxisome Proliferator-Activated Receptor α
CB: Calculus Bovis
AST: Aspartate aminotransferase
ALT: Alanine aminotransferase
ALP: Alkaline phosphatase
TBIL: Total bilirubin
DBIL: Direct bilirubin
GSH: Glutathione
TCHO: Total cholesterol
TG: Triglyceride
HDLC: High-density lipoprotein cholesterol
LDLC: Low-density lipoprotein cholesterol
MDA: Malondialdehyde
FFA: Free fatty acid
HYP: Hydroxyproline
HPLC: High-performance liquid chromatography
LC–MS/MS: Liquid chromatography-tandem mass spectrometry
TCA: Taurocholate
TDCA: Taurodesoxycholate
GCA: Glycocholic acid
GDCA: Glycodesoxycholic acid
CA: Cholic acid
CDCA: Chenodeoxycholic acid
SPF: Specific-pathogen-free
CMC-Na: Sodium carboxymethyl cellulose
SD: Sprague–Dawley
HepG2: Human hepatocellular carcinoma cells
Caco-2: Human colorectal adenocarcinoma cells
ATCC: The American Type Culture Collection
DMEM/F-12: Dulbecco’s Modified Eagle Medium/Nutrient Mixture F-12
FBS: Fetal bovine serum
P/S: Penicillin/streptomycin
NEAA: Non-essential amino acids
DCA: Deoxycholic acid
HDCA: Hyodeoxycholic acid
H&E: Hematoxylin and Eosin
ESI: Electrospray ionization
GO: Gene Ontology
KEGG: Kyoto Encyclopedia of Genes and Genomes
GSEA: Gene set enrichment analysis
PVDF: Polyvinylidene difluoride
SEM: Standard error of the mean
ANOVA: One-way analysis of variance
DEGs: Differentially expressed genes
PPI: Protein–protein interaction
PCA: Principal component analysis
NRs: Nuclear receptors
