# Supplementary material for: Calculus Bovis ameliorates primary sclerosing cholangitis via a dual-pronged mechanism restoring bile acid and lipid homeostasis in the gut-liver axis
Source: Chin Med. 2026 Jun 8;21:165. doi: 10.1186/s13020-026-01441-w (PMC13244648; doi:10.1186/s13020-026-01441-w)
Supplement: Supplementary file 1 — Supplementary Material 1 [file 13020_2026_1441_MOESM1_ESM.docx]

***Calculus Bovis* ameliorates primary sclerosing cholangitis via a dual-pronged mechanism restoring bile acid and lipid homeostasis in the gut-liver axis**

Xuepeng Gong^1†^, Yufei Chen^1†^, Tinghui Zhao^2^, Ninghong Li^3^, Guangjie Yang^1^, Lihui Qiu^1^, Zaoqin Yu^1^, Dong Liu^1*^, Dong Xiang^1*^

^1^Department of Pharmacy, Tongji Hospital, Tongji Medical College, Huazhong University of Science and Technology, Wuhan Hubei 430030, China

^2^Department of Pharmacy, Wuhan Mental Health Center, Wuhan Hubei 430030, China

^3^Department of Pharmacy, The Third Affiliated Hospital of Nanchang University, Nanchang Jiangxi 330008, China

* Correspondence to:

Dong Xiang: xiangdong@tjh.tjmu.edu.cn

Dong Liu: [ld2069@outlook.com](mailto:ld2069@outlook.com)

Xuepeng Gong: xue-penggong@tjh.tjmu.edu.cn

^†^These authors contributed equally to this work.

**Supplementary figures**

**
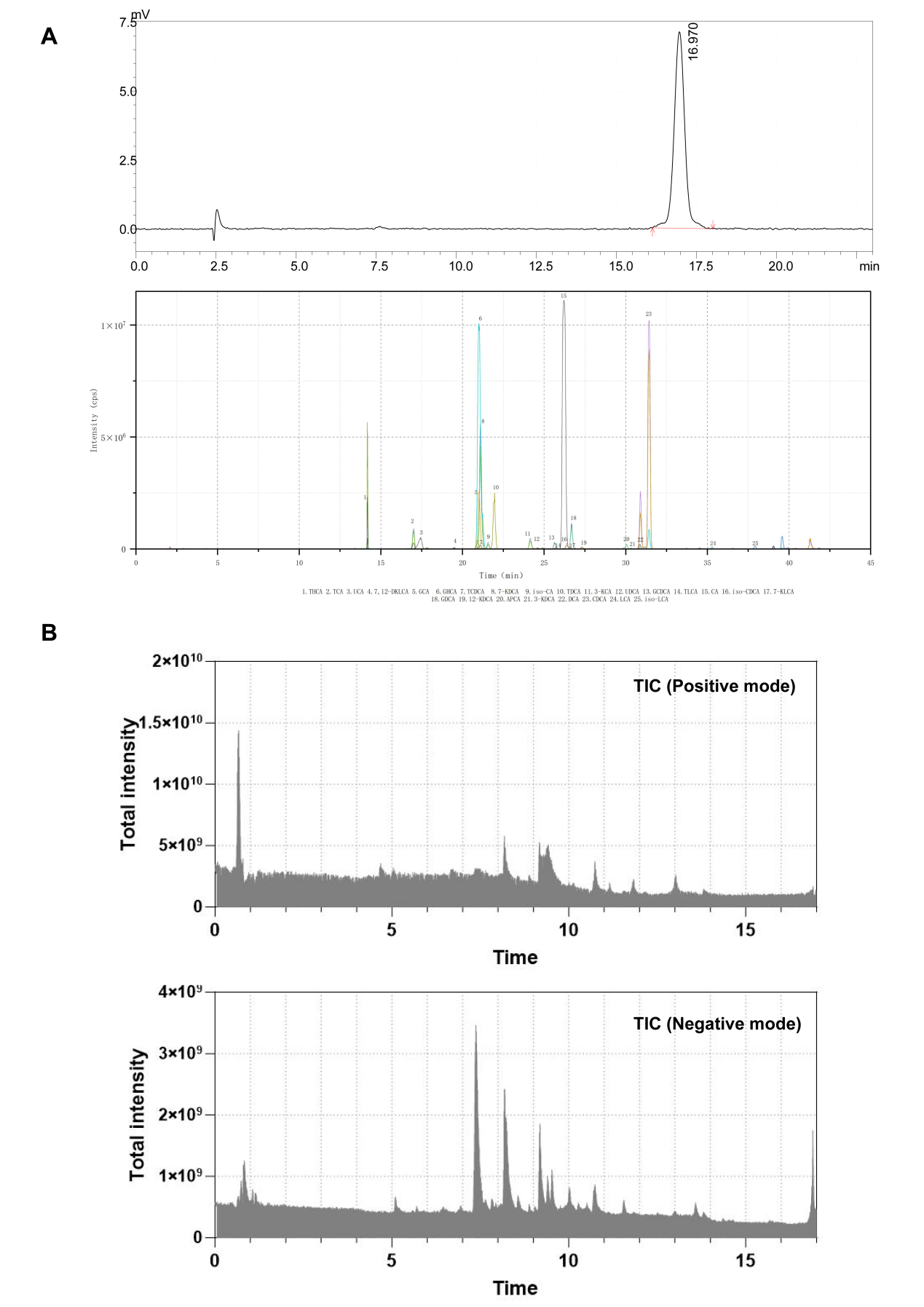
**

**Fig. S1. Quality control and chemical characterization of *Calculus Bovis* (CB).**

1. Representative HPLC chromatogram for bilirubin quantification (upper) and LC-MS/MS base peak chromatogram for bile acid analysis (lower) of the CB material used. (B) UHPLC-Q-Orbitrap MS total ion (positive and negative modes) chromatogram of CB. Major peaks were tentatively identified as various BAs and amino acids (see Supplementary Table S2 for details).

**
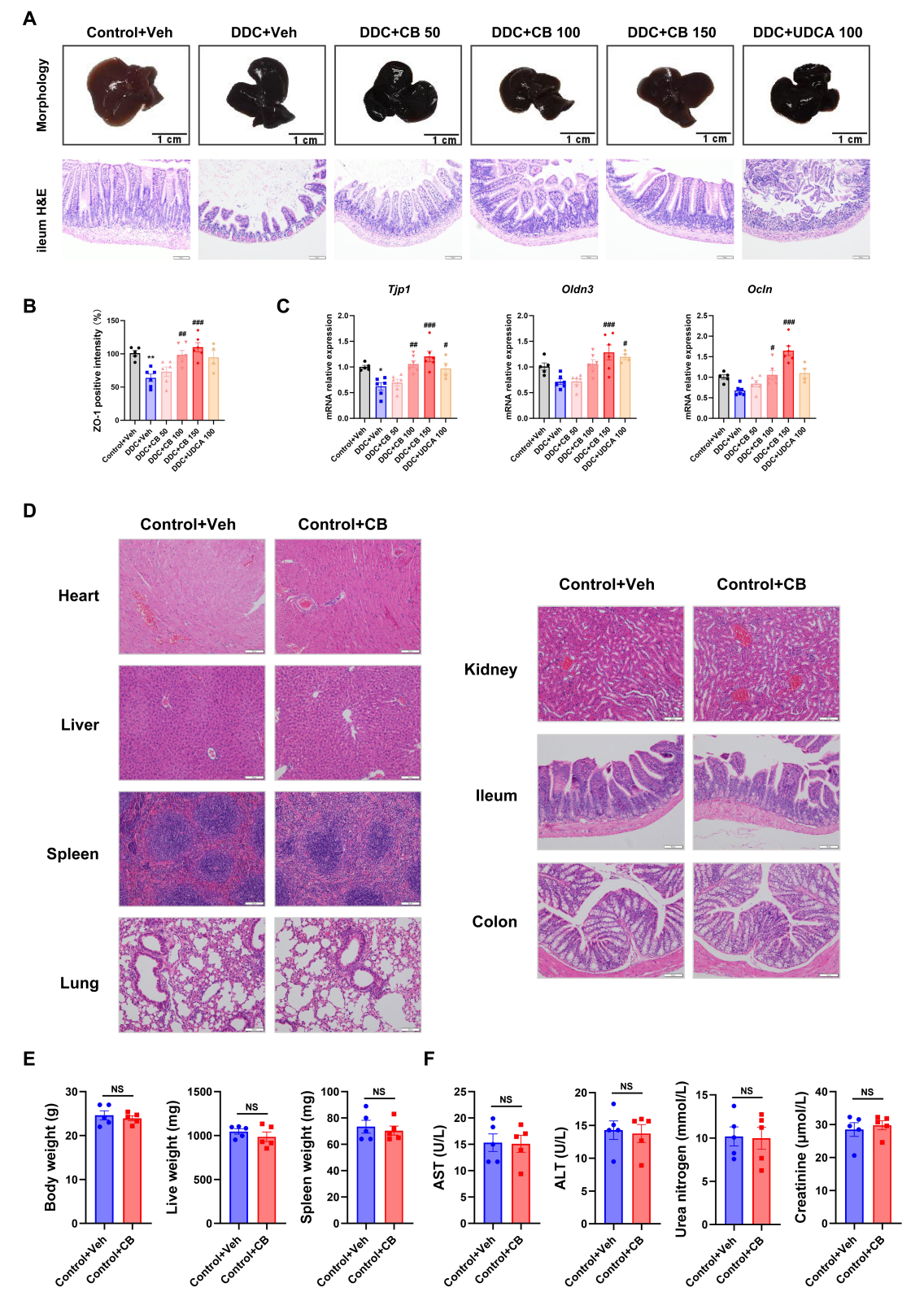
**

**Fig. S2. Supplementary histopathological data and *in vivo* safety evaluation of CB.**

(A) Representative photographs of liver morphology (upper) and H&E-stained sections of ileum (lower) from the indicated groups. (B) Quantification of ZO-1 positive area in ileum (n=4). (C) Ileal mRNA levels of tight junction-related genes (*Tjp1*, *Ocln*, *Oldn3*) normalized to *Hprt1* (n=6). (D) Representative H&E-stained sections of the heart, liver, spleen, lung, kidney, ileum, and colon from normal mice treated with high-dose CB (150 mg/kg/day) for 28 days, showing no histopathological abnormalities. (E) Body weight, liver weight, and spleen weight of normal mice after 28-day CB treatment. (F) Serum levels of AST, ALT, urea nitrogen, and creatinine in normal mice after CB treatment, indicating no hepatic or renal toxicity. Data are presented as mean ± SEM. Scale bar: 100 μm. *p < 0.05, **p < 0.01, ***p < 0.001 vs. DDC+Veh; #p < 0.05, ##p < 0.01, ###p < 0.001 vs. Control+Veh.

**
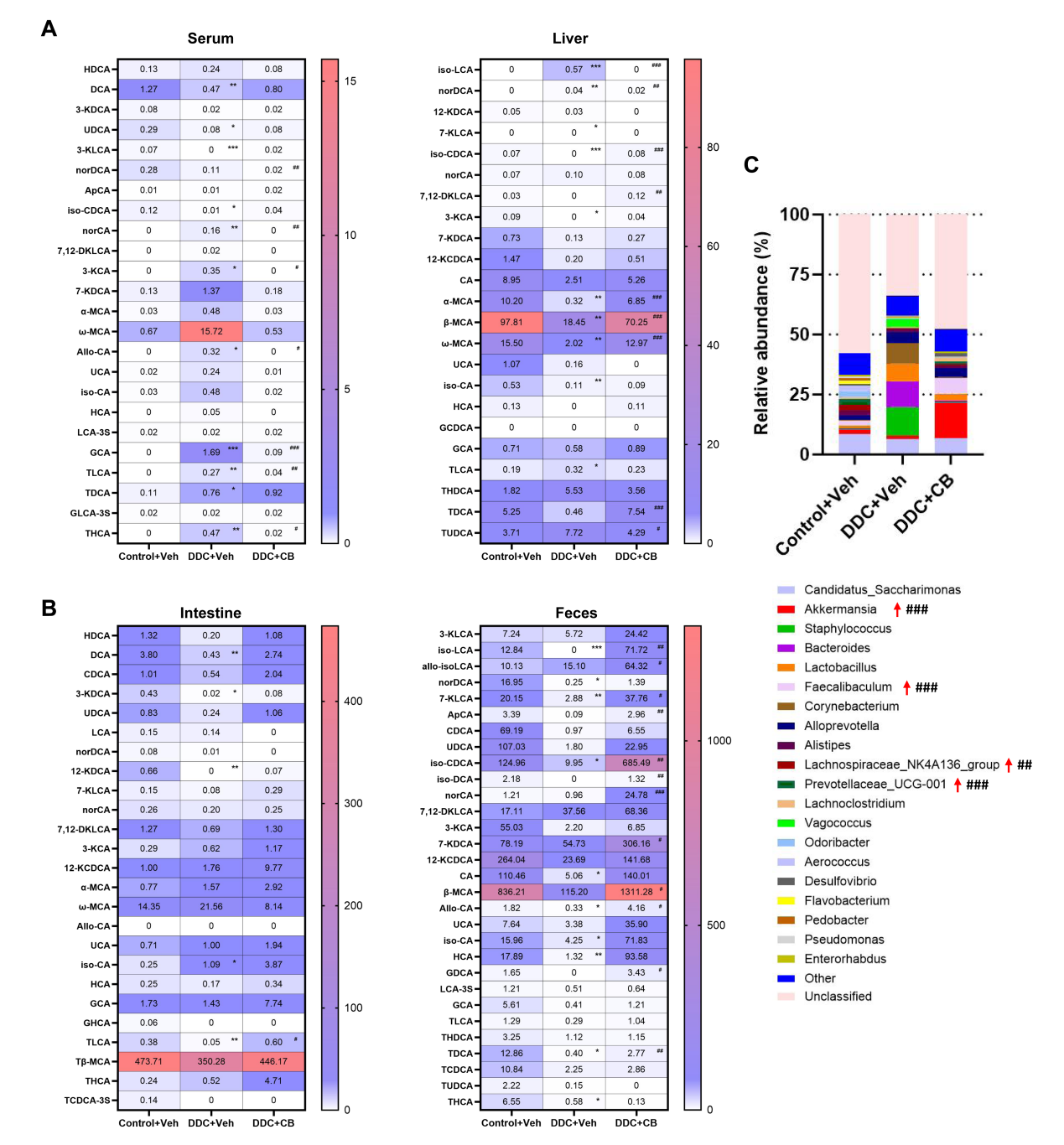
**

**Fig. S3. CB modulates individual bile acid profiles and gut microbiota composition in the enterohepatic circulation.**

(A, B) Heatmap visualization of the concentrations of individual BAs in (A) serum and liver, and (B) intestine and feces across the Control+Veh, DDC+Veh, and DDC+CB (150 mg/kg) groups. (C) Alterations in the relative abundance of the top 20 bacterial genera in fecal samples among the three groups as determined by 16S rRNA gene sequencing. Data are presented as mean ± SEM (n=6). ^*^*p* < 0.05, ^**^*p* < 0.01, ^***^*p* < 0.001 *vs*. Control+Veh; ^#^*p* < 0.05, ^##^*p* < 0.01, ^###^*p* < 0.001 *vs*. DDC+Veh.


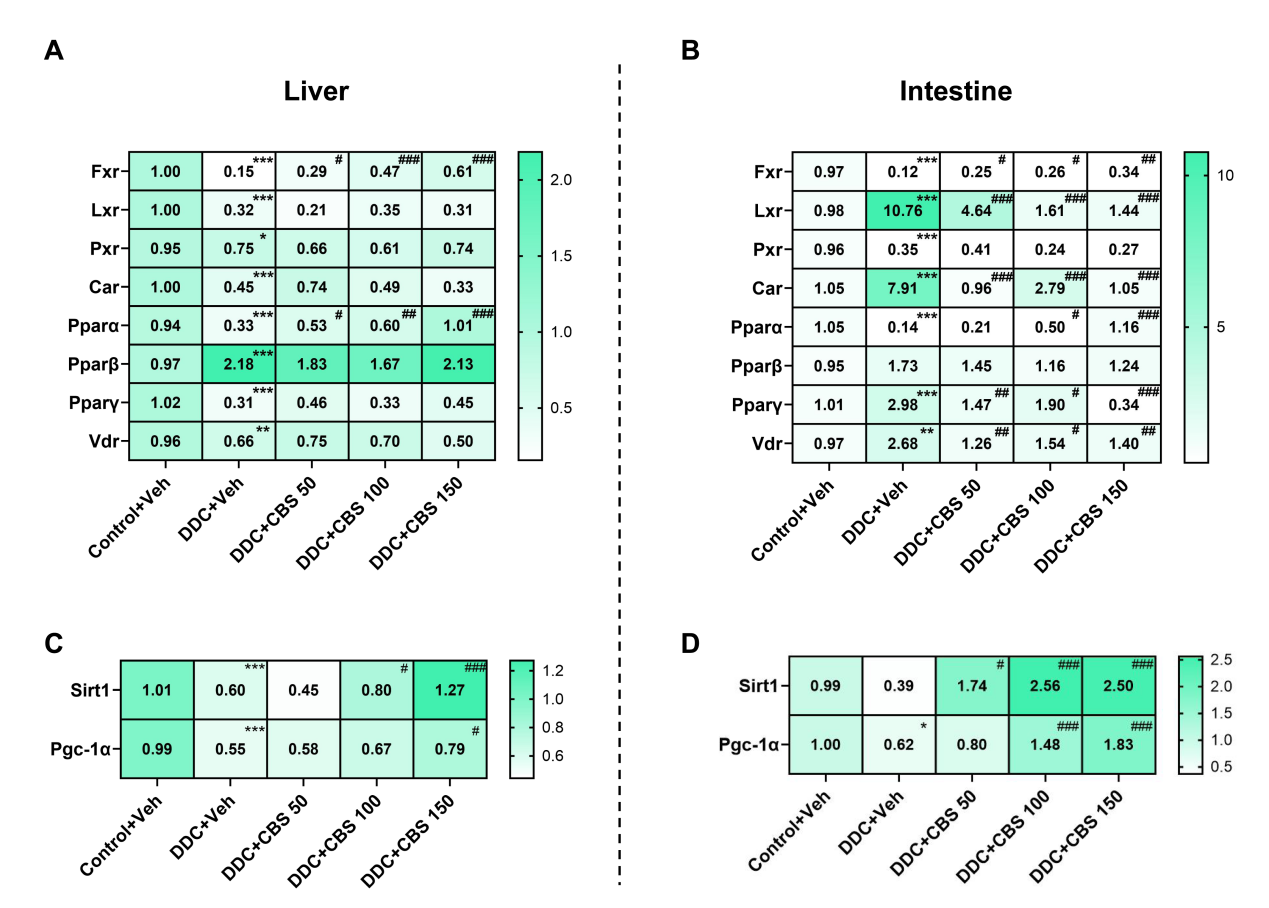


**Fig. S4. Quantitative analysis of nuclear receptor and SIRT1/PGC‑1α protein expression in liver and intestine.**

(A, B) Quantitative analysis of hepatic (A) and intestinal (B) nuclear receptor protein levels from Western blots. (C, D) Quantitative analysis of hepatic (C) and intestinal (D) SIRT1 and PGC-1α protein levels from Western blots. All values were normalized to Gapdh and presented as mean ± SEM (n=4-6). *p < 0.05, **p < 0.01, ***p < 0.001 vs. Control+Veh; #p < 0.05, ##p < 0.01, ###p < 0.001 vs. DDC+Veh.

**
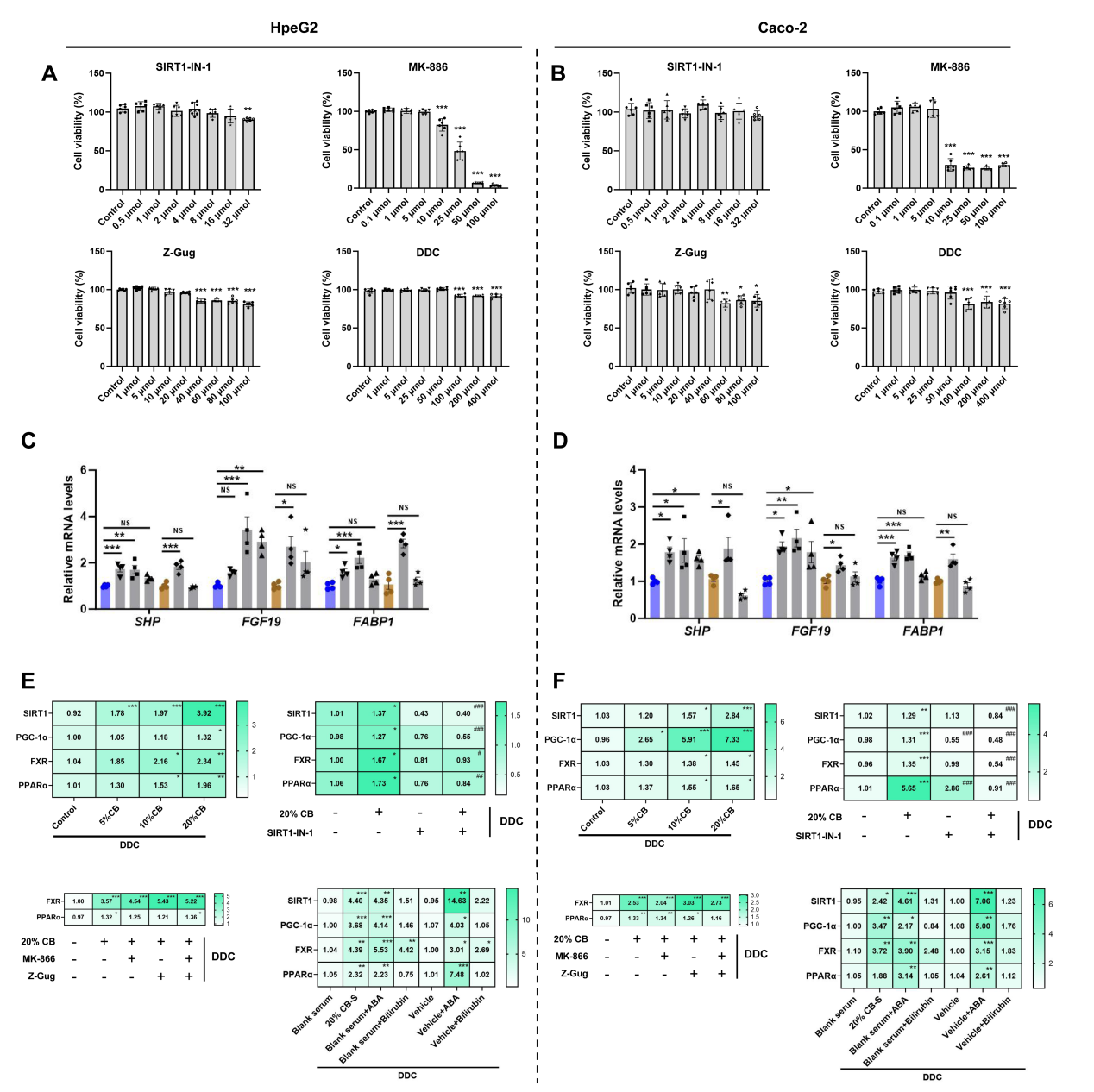
**

**Fig. S5. In vitro cytotoxicity assay and supplementary cell experiment data.**

(A, B) Cell viability of HepG2 (A) and Caco-2 (B) cells treated with DDC (1-400 μM), SIRT1-IN-1 (1-80 μM), MK-886 (0.1-100 μM), or Z-guggulsterone (Z-gug, 1-100 μM) for 24 hours, as determined by CCK-8 assay. (C, D) RT-qPCR analysis of the indicated genes in HepG2 (C) and Caco-2 (D) cells treated with the artificial BA mixture (ABA) or equimolar bilirubin, either supplemented into control serum (Con-S) or applied directly in serum-free medium. (C, D) Quantification of SIRT1, PGC-1α, FXR, and PPARα protein levels in HepG2 (C) and Caco-2 (D) cells from the experiments presented in Fig. 7B-S. Data are presented as mean ± SEM (n=3-4 independent experiments). *p < 0.05, **p < 0.01, ***p < 0.001 vs. the corresponding control (Con-S or Veh) group; #p < 0.05, ##p < 0.01, ###p < 0.001 vs. the CB-S alone group.


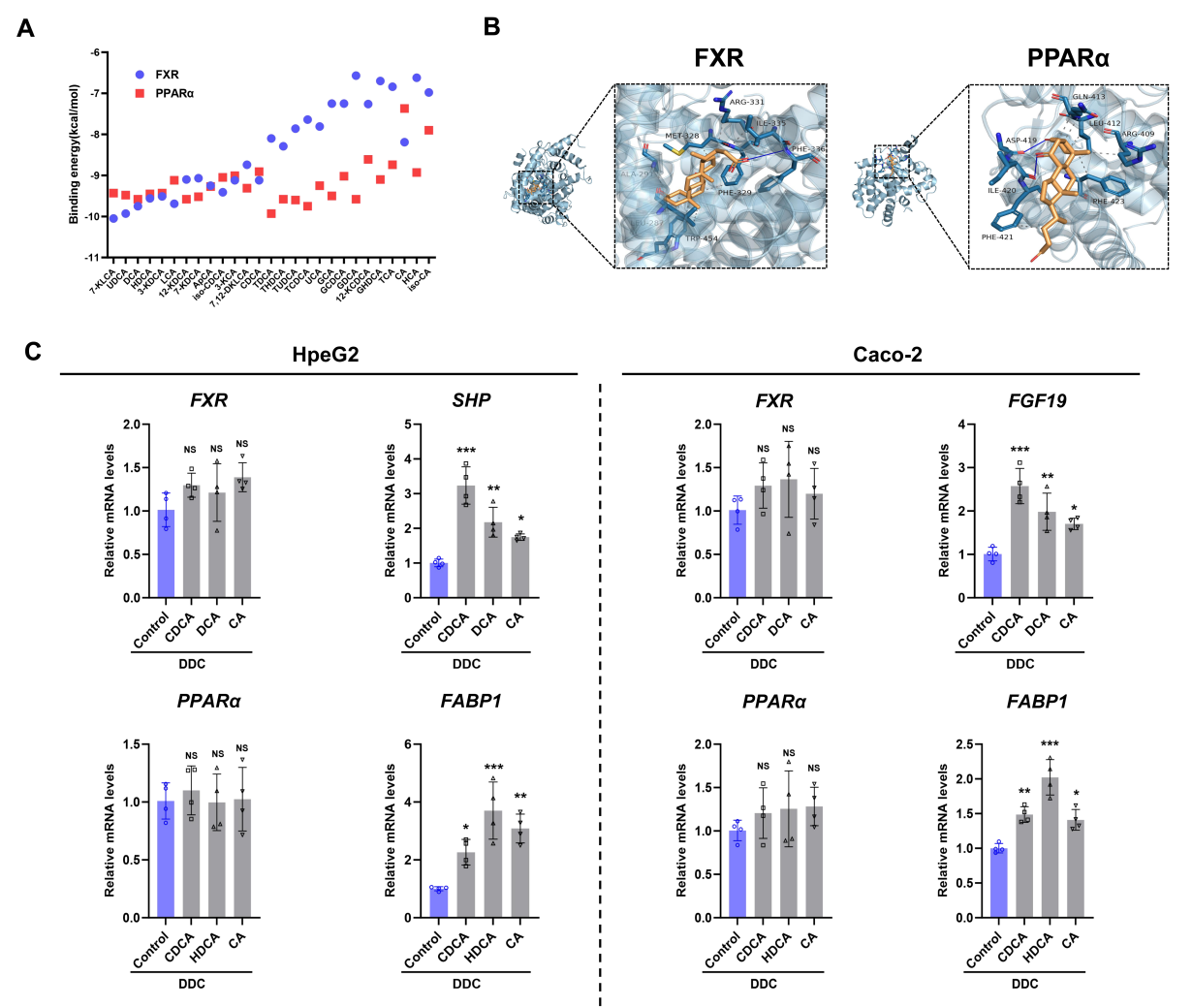


**Fig. S6. Molecular docking of blood‑entry BAs from CB with FXR and PPARα, and *in vitro* validation of direct receptor activation.**

(A) Predicted binding energies (kcal/mol) of the major blood‑entry BA components of CB docked into the ligand‑binding pockets of human FXR (PDB: 3BEJ) and PPARα (PDB: 6KAX). (B) Representative docking poses of CDCA (yellow sticks) within the binding pockets of FXR (left) and PPARα (right). Hydrogen bonds are indicated by blue dashed lines. (C) mRNA expression of *FXR*, *PPARα,* and their target genes in HepG2 (*SHP*, *FABP1*) and Caco‑2 (*FGF19*, *FABP1*) cells after 24‑h treatment with individual BAs (50 μM) (n=4). Data are presented as mean ± SEM. *p < 0.05, **p < 0.01, ***p < 0.001 vs. Vehicle group. Abbreviations: CA, cholic acid; CDCA, chenodeoxycholic acid; DCA, deoxycholic acid; HDCA, hyodeoxycholic acid.

**Supplementary Tables**

Supplementary Table S1.

Quantification of bile acids in *Calculus Bovis* (CB), drug-containing serum (CB-S), and control serum (Con-S), with calculation of the artificial bile acid mixture (ABA) composition

| Bile acid compound | *Calculus Bovis* | Blood entered? | CB-S | Con-S | ABA mixture (CB-S-Con-S) |
| --- | --- | --- | --- | --- | --- |
|  | mg/g | (Y/N) | μM | μM | μM |
| HDCA | - | Y | 21.8 | 1.76 | **20.04** |
| DCA | 4.131 | Y | 16.1 | 0.597 | **15.503** |
| CA | 11.102 | Y | 18.2 | 3.28 | **14.92** |
| CDCA | 1.314 | Y | 13.06 | 0.732 | **12.328** |
| 12-KCDCA | 0.405 | Y | 5.33 | 0.0355 | **5.2945** |
| TCA | 8.813 | Y | 4.66 | 0.3537 | **4.3063** |
| GCA | 96.46 | Y | 4.379 | 0.1503 | **4.2287** |
| 7-KDCA | 2.952 | Y | 2.81 | 0.086 | **2.724** |
| 12-KDCA | 0.133 | Y | 2.341 | 0.0348 | **2.3062** |
| LCA | 0.133 | Y | 2.1788 | 0.0509 | **2.1279** |
| iso-CA | 0.586 | Y | 2.13 | 0.00583 | 2.12417 |
| 7,12-DKLCA | 0.012 | Y | 1.435 | 0.0256 | 1.4094 |
| 3-KCA | 0.232 | Y | 0.934 | 0.0104 | 0.9236 |
| UCA | 4.086 | Y | 0.9836 | 0.098 | 0.8856 |
| TUDCA | 0.026 | Y | 0.635 | 0 | 0.635 |
| GDCA | 17.532 | Y | 0.632 | 0.0104 | 0.6216 |
| iso-CDCA | 0.076 | Y | 0.715 | 0.0946 | 0.6204 |
| 7-KLCA | 0.093 | Y | 0.708 | 0.177 | 0.531 |
| TDCA | 7.394 | Y | 0.4133 | 0.0588 | 0.3545 |
| ApCA | 0.011 | Y | 0.3603 | 0.00647 | 0.35383 |
| 3-KDCA | - | Y | 0.324 | 0.0303 | 0.2937 |
| HCA | - | Y | 0.236 | 0.00275 | 0.23325 |
| UDCA | 0.062 | Y | 0.27 | 0.0612 | 0.2088 |
| GHDCA | - | Y | 0.209 | 0.0227 | 0.1863 |
| THDCA | 0.014 | Y | 0.5781 | 0.4271 | 0.151 |
| GCDCA | 4.911 | Y | 0.143 | 0.00505 | 0.13795 |
| TCDCA | 3.572 | Y | 0.1121 | 0 | 0.1121 |
| all-isoLCA | 0.522 | N | 0 | 0 | 0 |
| allo-CA | - | N | 0 | 0 | 0 |
| DHCA | - | N | 0 | 0 | 0 |
| GCDCA-3S | - | N | 0 | 0 | 0 |
| GHCA | 37.1 | N | 0 | 0 | 0 |
| GLCA | 0.096 | N | 0 | 0 | 0 |
| GLCA-3S | - | N | 0 | 0 | 0 |
| GUDCA | 0.062 | N | 0 | 0 | 0 |
| iso-DCA | - | N | 0 | 0 | 0 |
| LCA-3S | 0.015 | N | 0 | 0 | 0 |
| nor-CA | 0.017 | N | 0 | 0 | 0 |
| norDCA | 0.005 | N | 0 | 0 | 0 |
| TCDCA-3S | - | N | 0 | 0 | 0 |
| THCA | 8.287 | N | 0 | 0 | 0 |
| TLCA | 0.465 | N | 0 | 0 | 0 |
| TLCA-3S | - | N | 0 | 0 | 0 |
| Tbeta-MCA | - | N | 0.1424 | 0.16529 | -0.02289 |
| 3-KLCA | - | N | 0.18 | 0.21 | -0.03 |
| Talpha-MCA | - | N | 0.07579 | 0.1477 | -0.07191 |
| Iso-LCA | - | N | 0.11 | 0.182 | -0.072 |
| omega-MCA | - | N | 1.25 | 1.5862 | -0.3362 |
| alpha-MCA | - | N | 1.17 | 1.508 | -0.338 |
| beta-MCA | - | N | 1.11 | 1.827 | -0.717 |

Notes: Y, Yes; N, No. Full name of the abbreviation for BA: see Supporting Table 3. The ABA concentration was calculated as [CB-S] - [Con-S]. The ten BAs with the highest positive net concentrations (shown in bold) were used to prepare the ABA. Negative values represent BAs where Con-S levels exceeded CB-S levels and were not included in the mixture.

Supplementary Table S2.

Identifcation of the chemical ingredients in CB

| **NO.** | **Metabolite** | **Formula** | **Retention time(min)** | **m/z** | **Mode** | **Adducts** |
| --- | --- | --- | --- | --- | --- | --- |
| 1 | L-Arginine | C6H14N4O2 | 0.76 | 173.10 | neg | M-H |
| 2 | Citrulline | C6H13N3O3 | 0.78 | 175.12 | pos | M+NH4-H2O |
| 3 | 2'-Deoxyadenosine | C10H13N5O3 | 0.78 | 324.13 | pos | M+CH3OH+Na+H2O |
| 4 | N-Acetyl-L-alanine | C5H9NO3 | 0.79 | 130.05 | neg | M-H |
| 5 | L-Aspartic acid | C4H7NO4 | 0.79 | 132.03 | neg | M-H |
| 6 | Alanine | C3H7NO2 | 0.80 | 90.06 | pos | [M+H]+ |
| 7 | L-Threonine | C4H9NO3 | 0.80 | 120.07 | pos | M+H |
| 8 | L-Asparagine | C4H8N2O3 | 0.80 | 133.06 | pos | M+H |
| 9 | L-Carnitine | C7H15NO3 | 0.80 | 162.11 | pos | M+H |
| 10 | L-Glutamic acid | C5H9NO4 | 1.05 | 130.05 | pos | M+H-H2O |
| 11 | alpha-Dihydroartemisinin | C15H24O5 | 1.06 | 348.18 | pos | M+CH3CN+Na |
| 12 | Tyrosine | C9H11NO3 | 1.08 | 182.08 | pos | [M+H]+ |
| 13 | Guanosine | C10H13N5O5 | 1.08 | 284.10 | pos | M+H |
| 14 | Inosine | C10H12N4O5 | 1.08 | 267.07 | neg | M-H |
| 15 | L-Leucine | C6H13NO2 | 1.13 | 132.10 | pos | M+H |
| 16 | Adenosine | C10H13N5O4 | 1.46 | 268.10 | pos | M+H |
| 17 | Isoleucine | C6H13NO2 | 2.56 | 132.10 | pos | [M+H]+ |
| 18 | 5,7-Dimethoxy-2H-chromen-2-one | C11H10O4 | 3.91 | 207.07 | pos | [M+H]+ |
| 19 | 2-Hydroxyadenosine | C10H12N5O5 | 4.62 | 305.07 | pos | M+Na, M+K |
| 20 | Linderaspirone A | C34H32O10 | 4.79 | 691.21 | neg | M+HCOOH+HCOO |
| 21 | Decanoic Acid | C10H20O2 | 4.81 | 190.18 | pos | M+NH4 |
| 22 | 2-Hydroxy-4-methoxybenzaldehyde | C8H8O3 | 5.83 | 153.05 | pos | M+H |
| 23 | Coumarin | C9H6O2 | 6.18 | 147.04 | pos | [M+H]+ |
| 24 | Echioidinin | C16H12O5 | 6.90 | 283.06 | neg | M-H |
| 25 | 3-Dehydrocholic acid | C24H38O5 | 8.09 | 405.26 | neg | [M-H]- |
| 26 | Tauro-Obeticholic acid | C28H49NO6S | 8.12 | 558.35 | neg | M-H+CH3OH |
| 27 | glycocholic acid | C26H43NO6 | 8.18 | 466.32 | pos | M+H |
| 28 | glycoursodeoxycholic acid | C26H43NO5 | 8.24 | 448.31 | neg | M-H |
| 29 | Taurochenodeoxycholic Acid | C26H45NO6S | 8.25 | 997.58 | neg | 2M-H |
| 30 | Sodium taurochenodeoxycholate | C26H44NNaO6S | 8.28 | 580.29 | neg | M+CH3COO |
| 31 | Taurocholic acid | C26H45NO7S | 8.99 | 554.25 | pos | M+K |
| 32 | Cholic Acid | C24H40O5 | 9.21 | 448.31 | neg | M-H+CH3CN |
| 33 | Myristoyl Ethanolamide | C16H33NO2 | 9.46 | 272.26 | pos | [M+H]+ |
| 34 | Deoxycholic acid (Choleic acid | C24H40O4 | 9.88 | 391.28 | neg | M-H |
| 35 | Palmitoleoyl Ethanolamide | C18H35NO2 | 9.89 | 298.27 | pos | [M+H]+ |
| 36 | Cucurbitacin E | C32H44O8 | 9.99 | 579.29 | pos | M+Na |
| 37 | pseudolaric acid B | C23H28O8 | 10.46 | 433.18 | pos | M+H |
| 38 | Ursodeoxycholic acid | C24H40O4 | 10.71 | 427.26 | neg | M+Cl |
| 39 | Glycolithocholic acid | C26H43NO4 | 10.83 | 466.35 | pos | M+CH3OH+H |
| 40 | 3-Amino-3-(hydroxymethyl)-1-(4-octylphenyl)-1,4-butanediol | C19H33NO3 | 10.98 | 306.24 | pos | M+H-H2O |
| 41 | Oleoylethanolamide | C20H39NO2 | 11.01 | 343.33 | pos | M+NH4 |
| 42 | 9,17-Octadecadiene-12,14-diyne-1,11,16-triol | C18H26O3 | 11.49 | 323.22 | pos | M+CH3OH+H |
| 43 | Linolelaidic acid | C18H32O2 | 11.77 | 263.24 | pos | M+H-H2O |
| 44 | Tyramine | C8H11NO | 11.83 | 121.06 | pos | [M+H-NH3]+ |
| 45 | Palmitoleic acid | C16H30O2 | 12.22 | 271.23 | neg | M-H+H2O |
| 46 | Eicosapentaenoic Acid | C20H30O2 | 12.46 | 366.24 | pos | M+CH3CN+Na |
| 47 | Cannabichromene | C21H30O2 | 12.51 | 355.23 | pos | M+H2O+Na |
| 48 | Glyceryl monolinoleate | C21H38O4 | 12.81 | 372.31 | pos | M+NH4 |
| 49 | Dehydrophytosphingosine (not validated) - 2H | C18H35NO3 | 12.99 | 314.27 | pos | [M+H]+ |
| 50 | D-Erythro-Sphingosine | C18H37NO2 | 13.07 | 300.29 | pos | M+H |
| 51 | Chonemorphine | C23H42N2 | 13.33 | 397.38 | pos | M+H2O+CH3OH+H |
| 52 | N-Benzyllinoleamide | C25H39NO | 13.58 | 410.31 | pos | M+H2O+Na |
| 53 | Arachidoyl Ethanolamide | C22H45NO2 | 13.60 | 356.35 | pos | [M+H]+ |
| 54 | Arachidonic acid | C20H32O2 | 13.64 | 303.23 | neg | M-H |
| 55 | Phytosphingosine | C18H39NO3 | 13.71 | 381.31 | pos | M+CH3CN+Na |
| 56 | Palmitic Acid | C16H32O2 | 14.36 | 315.25 | neg | M+CH3COO |
| 57 | Stearic acid | C18H36O2 | 15.69 | 343.28 | neg | M+CH3COO |
| 58 | Betaine | C5H11NO2 | 16.90 | 118.09 | pos | M+H |
| 59 | Isovaleramide | C5H11NO | 16.91 | 120.10 | pos | M+H2O+H |
| 60 | Citric acid | C6H8O7 | 16.92 | 191.02 | neg | M-H |

Supplementary Table S3.

Information of all bile acids

| Bile acids | Abbreviation | MRM Transition (m/z) | Source |
| --- | --- | --- | --- |
| 3-Dehydrolithocholic acid | 3-KLCA | 373.4/373.4 | Cayman Chemical |
| Lithocholic Acid | LCA | 375.4/375.4 | Zzstandard |
| Isolithocholic Acid | iso-LCA | 375.4/375.4 | Cayman Chemical |
| Isoallolithocholic acid | allo-isoLCA | 375.4/375.4 | Cayman Chemical |
| Nordeoxycholic Acid | norDCA | 377.4/377.4 | Zzstandard |
| 3-Ketodeoxycholic Acid | 3-KDCA | 389.4/389.4 | Cayman Chemical |
| 12-Ketodeoxycholic Acid | 12-KDCA | 389.4/389.4 | Sigma |
| 7-Ketolithocholic Acid | 7-KLCA | 389.4/389.4 | Cayman Chemical |
| Apocholic Acid | ApCA | 389.4/389.4 | Zzstandard |
| Deoxycholic Acid | DCA | 391.3/391.3 | IsoReag |
| Chenodeoxycholic Acid | CDCA | 391.3/391.3 | NIFDC |
| Ursodeoxycholic Acid | UDCA | 391.3/391.3 | NIFDC |
| Hyodeoxycholic Acid | HDCA | 391.3/391.3 | NIFDC |
| Isochenodeoxycholic Acid | iso-CDCA | 391.3/391.3 | Zzstandard |
| Isodeoxycholic Acid | iso-DCA | 391.3/391.3 | Cayman Chemical |
| Norcholic Acid | norCA | 393.4/393.4 | Zzstandard |
| Dehydrocholic Acid | DHCA | 401.1/249.2 | Zzstandard |
| 7,12-Diketolithocholic Acid | 7,12-DKLCA | 403.4/403.4 | Cayman Chemical |
| 3-Ketocholic Acid | 3-KCA | 405.4/405.4 | Cayman Chemical |
| 7-Ketodeoxycholic Acid | 7-KDCA | 405.4/405.4 | Zzstandard |
| 12-Ketochenodeoxycholic Acid | 12-KCDCA | 405.4/405.4 | Zzstandard |
| Cholic Acid | CA | 407.3/407.3 | NIFDC |
| α-Muricholic Acid | α-MCA | 407.3/407.3 | Zzstandard |
| β-Muricholic Acid | β-MCA | 407.3/407.3 | Zzstandard |
| ω-Muricholic Acid | ω-MCA | 407.3/407.3 | Zzstandard |
| Allocholic Acid | Allo-CA | 407.3/407.3 | Cayman Chemical |
| Ursocholic acid | UCA | 407.3/407.3 | Cayman Chemical |
| Isocholic Acid | iso-CA | 407.3/407.3 | Cayman Chemical |
| Hyocholic Acid | HCA | 407.3/407.3 | Zzstandard |
| Glycolithocholic Acid | GLCA | 432.4/74.0 | Zzstandard |
| Glycochenodeoxycholic Acid | GCDCA | 448.2/74.0 | Zzstandard |
| Glycoursodeoxycholic Acid | GUDCA | 448.2/74.0 | Zzstandard |
| Glycohyodeoxycholic Acid | GHDCA | 448.2/74.0 | Cayman Chemical |
| Glycodeoxycholic Acid | GDCA | 448.3/74.0 | Zzstandard |
| Lithocholic Acid 3-Sulfate | LCA-3S | 455.3/97.0 | Zzstandard |
| Glycocholic Acid | GCA | 464.5/74.0 | Zzstandard |
| Glycohyocholic Acid | GHCA | 464.5/74.0 | Cayman Chemical |
| Taurolithocholic Acid | TLCA | 482.3/80.0 | Zzstandard |
| Taurohyodeoxycholic Acid | THDCA | 498.3/80.0 | Cayman Chemical |
| Taurodeoxycholic Acid | TDCA | 498.3/80.0 | Cayman Chemical |
| Taurochenodeoxycholic Acid | TCDCA | 498.3/80.0 | Cayman Chemical |
| Tauroursodeoxycholic Acid | TUDCA | 498.3/80.0 | Cayman Chemical |
| Glycolithocholic Acid 3-Sulfate | GLCA-3S | 512.3/432.3 | IsoReag |
| Taurocholic Acid | TCA | 514.3/80.0 | Cayman Chemical |
| Tauro-α-muricholic Acid | Tα-MCA | 514.3/80.0 | Zzstandard |
| Tauro-β-muricholic Acid | Tβ-MCA | 514.3/80.0 | Zzstandard |
| Taurohyocholic Acid | THCA | 514.3/80.0 | Cayman Chemical |
| Glycochenodeoxycholic Acid 3-Sulfate | GCDCA-3S | 528.2/448.3 | lsoReag |
| Taurolithocholic Acid 3-Sulfate | TLCA-3S | 562.3/482.4 | Zzstandard |
| Taurochenodeoxycholic acid 3-sulfate | TCDCA-3S | 578.2/498.3 | lsoReag |

Notes: NIFDC, the National Institute for Food and Drug Control.

Supplementary Table S4.

Information on antibodies

| Antibodies | Dilution ratio | Source | Item numbers |
| --- | --- | --- | --- |
| GAPDH | 1:5000 | Proteintech | 10494-1-AP |
| CYP7A1 | 1:2000 | Proteintech | 18054-1-AP |
| BAAT | 1:800 | Servicebio | GB111858 |
| BSEP | 1:10000 | Proteintech | 67512-1-Ig |
| NTCP | 1:1000 | Absin | abs123885 |
| ASBT | 1:1000 | Absin | abs119604 |
| CPT1A | 1:5000 | Proteintech | 660393-3-Ig |
| ANGPTL4 | 1:4000 | Proteintech | 67577-1-Ig |
| FXR | 1:4000 | Proteintech | 25055-1-AP |
| LXR | 1:800 | Servicebio | GB114072 |
| PXR | 1:1000 | Beyotime | AF7845 |
| CAR | 1:800 | Servicebio | GB111418 |
| PPARα | 1:1000 | Beyotime | AF7794 |
| PPARβ | 1:1000 | Beyotime | AF7800 |
| PPARγ | 1:1000 | Proteintech | 81490-5-RR |
| VDR | 1:1000 | Beyotime | AF8316 |
| SIRT1 | 1:1000 | Beyotime | AF-0282 |
| PGC-1α | 1:5000 | Proteintech | 66369-1-Ig |
| Goat anti-rabbit HRP | 1:8000 | Proteintech | RGAR001 |
| Goat anti-mouse HRP | 1:8000 | Proteintech | RGAM001 |

Notes: The above dilution ratio is only used for western blotting experiments.

Supplementary Table S5.

List of primer sequences used in this study

| Primers | Species | Forward primer | Reverse primer |
| --- | --- | --- | --- |
| *Hprt1* | Mouse | AGTCCCAGCGTCGTGATTAG | TTTCCAAATCCTCGGCATAATGA |
| *Nos2* | Mouse | ACATCGACCCGTCCACAGTAT | CAGAGGGGTAGGCTTGTCTC |
| *Hmox1* | Mouse | AAGCCGAGAATGCTGAGTTCA | GCCGTGTAGATATGGTACAAGGA |
| *CK-19* | Mouse | GGGGGTTCAGTACGCATTGG | GAGGACGAGGTCACGAAGC |
| *Caspase-3* | Mouse | CTGACTGGAAAGCCGAAACTC | CGACCCGTCCTTTGAATTTCT |
| *Cyp7a1* | Mouse | GGGATTGCTGTGGTAGTGAGC | GGTATGGAATCAACCCGTTGTC |
| *Cyp8b1* | Mouse | CTAGGGCCTAAAGGTTCGAGT | GTAGCCGAATAAGCTCAGGAAG |
| *Cyp27a1* | Mouse | AGGGCAAGTACCCAATAAGAGA | TCGTTTAAGGCATCCGTGTAGA |
| *Cyp7b1* | Mouse | AACACCATTCCAGCTATGTTCTG | CCTCAAGAATAGTGCTTTCCAGG |
| *Cyp2c70* | Mouse | AGTATGGCCCTGTGTTTACTGT | GCCTTGGCTGGTTCTACTGAG |
| *Cyp3a11* | Mouse | CCGAGTGGATTTTCTTCAGC | GAGCCTCATCGATCTCATCC |
| *Baat* | Mouse | GTGCTGGTGGATTGATGGAGT | CCGAGGACCTTAGGATGTCTC |
| *Bacs* | Mouse | GTTCTCCCGTCCAAGACCATT | GCTCCGTACAGAGTGTAGCAAG |
| *Ugt1a1* | Mouse | GCTTCTTCCGTACCTTCTGTTG | GCTGCTGAATAACTCCAAGCAT |
| *Sult2a1* | Mouse | TAACTTACCCCAAGTCAGGAACG | ATGGGAAGATGGGAGGTTATGA |
| *Bsep* | Mouse | TCTGACTCAGTGATTCTTCGCA | CCCATAAACATCAGCCAGTTGT |
| *Mrp2* | Mouse | TCTTCGTCTCCTATGGTTTCCA | CGTGTGTTGAGTCGCTTGATT |
| *Ntcp* | Mouse | CAAACCTCAGAAGGACCAAACA | GTAGGAGGATTATTCCCGTTGTG |
| *Oatp1* | Mouse | GTGCATACCTAGCCAAATCACT | CCAGGCCCATAACCACACATC |
| *Mrp4* | Mouse | GGCACTCCGGTTAAGTAACTC | TGTCACTTGGTCGAATTTGTTCA |
| *Mrp3* | Mouse | CTGGGTCCCCTGCATCTAC | GCCGTCTTGAGCCTGGATAAC |
| *Asbt* | Mouse | TGATGTTTTCTATGGGGTGCAAT | TGAGAGGCATGATTCCAAACTG |
| *Ibabp* | Mouse | CTTCCAGGAGACGTGATTGAAA | AACTTGTTGCTCATAATGTTGCC |
| *Ostα* | Mouse | CCCTGACGGCATCTATGACC | TGGCTTGACGGAAAAGGATGG |
| *Ostβ* | Mouse | AGATGCGGCTCCTTGGAATTA | TGGCTGCTTCTTTCGATTTCTG |
| *Cd36* | Mouse | AGATGACGTGGCAAAGAACAG | CCTTGGCTAGATAACGAACTCTG |
| *Acsl1* | Mouse | TCTTGGTGTACTACTACGACGAT | CGAGAACCTAAACAAGGACCATT |
| *Cpt1a* | Mouse | CTATGCGCTACTCGCTGAAGG | GGCTTTCGACCCGAGAAGA |
| *Acox1* | Mouse | TCGAAGCCAGCGTTACGAG | GGTCTGCGATGCCAAATTCC |
| *Hmgcs2* | Mouse | GAAGAGAGCGATGCAGGAAAC | GTCCACATATTGGGCTGGAAA |
| *Hmgcr* | Mouse | AGCTTGCCCGAATTGTATGTG | TCTGTTGTGAACCATGTGACTTC |
| *Abcg5* | Mouse | CGCGAGACGTTGCGATACA | CTGCCAATCATTTGGTCCGC |
| *Abcg8* | Mouse | CTGTGGAATGGGACTGTACTTC | TGTTGTCACTTTCCGAGGAGA |
| *Npc1l1* | Mouse | CGCCCTTCTTTCTACATGGGT | GAATCTGCGCTTACGAGGGAG |
| *ApoA5* | Mouse | TCCTCGCAGTGTTCGCAAG | CGAAGCTGCCTTTCAGGTTCT |
| *Lpl* | Mouse | ATGGATGGACGGTAACGGGAA | CCCGATACAACCAGTCTACTACA |
| *Angptl4* | Mouse | CATCCTGGGACGAGATGAACT | TGACAAGCGTTACCACAGGC |
| *Fxr* | Mouse | GCTTGATGTGCTACAAAAGCTG | CGTGGTGATGGTTGAATGTCC |
| *Lxr* | Mouse | CTCAATGCCTGATGTTTCTCCT | TCCAACCCTATCCCTAAAGCAA |
| *Pxr* | Mouse | TAGGGACCTGCCTATTGAGGA | CCGTTTCCGTGTCGAACATC |
| *Car* | Mouse | ATATGGGCCGAGGAACTGTGT | GGCGTGGAAATGATAGCCTGT |
| *Pparα* | Mouse | TACTGCCGTTTTCACAAGTGC | AGGTCGTGTTCACAGGTAAGA |
| *Pparβ* | Mouse | GCAGCCTCAACATGGAATGTC | GAGCTTCATGCGGATTGTCC |
| *Pparγ* | Mouse | TCGCTGATGCACTGCCTATG | GAGAGGTCCACAGAGCTGATT |
| *Vdr* | Mouse | GTGCAGCGTAAGCGAGAGAT | GGATGGCGATAATGTGCTGTTG |
| *Fgf15* | Mouse | ATGGCGAGAAAGTGGAACGG | CTGACACAGACTGGGATTGCT |
| *Fabp1* | Mouse | ATGAACTTCTCCGGCAAGTACC | CTGACACCCCCTTGATGTCC |
| *Sirt1* | Mouse | ATGACGCTGTGGCAGATTGTT | CCGCAAGGCGAGCATAGAT |
| *Pgc-1α* | Mouse | TATGGAGTGACATAGAGTGTGCT | CCACTTCAATCCACCCAGAAAG |
| *GAPDH* | Human | ACAACTTTGGTATCGTGGAAGG | GCCATCACGCCACAGTTTC |
| *SIRT1* | Human | TGTGTCATAGGTTAGGTGGTGA | AGCCAATTCTTTTTGTGTTCGTG |
| *PGC-1α* | Human | TCTGAGTCTGTATGGAGTGACAT | CCAAGTCGTTCACATCTAGTTCA |
| *FXR* | Human | TGCAGATCAGACCGTGAATGA | TTGGTTGCCATTTCCGTCAAA |
| *PPARα* | Human | TTCGCAATCCATCGGCGAG | CCACAGGATAAGTCACCGAGG |
| *SHP* | Human | GTGCCCAGCATACTCAAGAAG | TGGGGTCTGTCTGGCAGTT |
| *FABP1* | Human | AAGACAGTGGTTCAGTTGGAAG | TGAGTTCGGTCACAGACTTGAT |
| *OSTβ* | Human | TCCAGGCAAGCAGAAAAGAAA | ACTGACAGCACATCTCTCTCT |
| *UGT1A1* | Human | TTGTCTGGCTGTTCCCACTTA | GGTCCGTCAGCATGACATCA |
| *FGF19* | Human | CGGAGGAAGACTGTGCTTTCG | CTCGGATCGGTACACATTGTAG |
| *IBABP* | Human | GCCCGCAACTTCAAGATCG | CCTTGCCAACAGTGAACTTGT |
